# Upregulation of RND3 Affects Trophoblast Proliferation, Apoptosis, and Migration at the Maternal-Fetal Interface

**DOI:** 10.3389/fcell.2020.00153

**Published:** 2020-03-13

**Authors:** Xiao-Ling Ma, Xiao Li, Fu-Ju Tian, Wei-Hong Zeng, Jun Zhang, Hui-Qin Mo, Shi Qin, Li-Qun Sun, Yu-Chen Zhang, Yan Zhang, Yi Lin

**Affiliations:** ^1^Shanghai Key Laboratory of Embryo Original Diseases, International Peace Maternity and Child Health Hospital, Shanghai Jiao Tong University School of Medicine, Shanghai, China; ^2^Department of Obstetrics and Gynecology, Shanghai Jiao Tong University Affiliated Sixth People’s Hospital, Shanghai, China; ^3^Department of Obstetrics and Gynecology, Renmin Hospital of Wuhan University, Wuhan, China

**Keywords:** RND3, FOXD3, recurrent miscarriage, trophoblast, ROCK, ERK1/2

## Abstract

Trophoblasts as the particular cells of the placenta play an important role in implantation and formation of the maternal-fetal interface. RND3 (also known as RhoE) is a unique member of the Rnd subfamily of small GTP-binding proteins. However, its function in cytotrophoblasts (CTBs) at the maternal-fetal interface is poorly understood. In the present study, we found that RND3 expression was significantly increased in trophoblasts from the villous tissues of patients with recurrent miscarriage (RM). RND3 inhibited proliferation and migration and promoted apoptosis in HTR-8/SVneo cells. Using dual-luciferase reporter and chromatin immunoprecipitation assays, we found that forkhead box D3 (FOXD3) is a key transcription factor that binds to the RND3 core promoter region and regulates RND3 expression. Here, the level of FOXD3 was upregulated in the first-trimester CTBs of patients with RM, which in turn mediated RND3 function, including inhibition of cell proliferation and migration and promotion of apoptosis. Further, we found that RND3 regulates trophoblast migration and proliferation via the RhoA-ROCK1 signaling pathway and inhibits apoptosis via ERK1/2 signaling. Taken together, our findings suggest that RND3 and FOXD3 may be involved in pathogenesis of RM and may serve as potential therapeutic targets.

## Introduction

Recurrent miscarriage (RM), defined as two or more consecutive spontaneous abortions, occurs in approximately 1–5% of all couples trying to conceive (Rai and Regan, 2006; Practice Committee of American Society for Reproductive Medicine, 2013). Numerous studies have shown that trophoblasts as the particular cells of the placenta play an important role in implantation and formation of the maternal-fetal interface (Red-Horse et al., 2004). The special structure of maternal-fetal interface is composed of differentiation of villous cytotrophoblast (CTB) cells at the tips of anchoring villi and migrating into the decidual tissue (Yilmaz and Christofori, 2009). Impaired trophoblast function is related to RM, intrauterine growth retardation, preeclampsia (PE), and many other pregnancy-related complications. Although trophoblast function appears to be tightly regulated, the mechanisms underlying trophoblast migration and apoptosis are complex and unknown.

The members of the Rho family of GTPases as a subfamily of the Ras family are included in the regulation of cellular functions and the basics of cellular biology, such as cell adhesion, proliferation, migration and survival, and play a critical role in tumor and other normal cells (Jaffe and Hall, 2005). There are 22 mammalian genes encoding Rho GTPases and they are divided into eight subgroup (Aspenström and Fransson, 2004). Most members of the Rho family can cycle by the ratio of an active, GTP-bound conformation and an inactive, GDP-bound conformation, and GDP/GTP exchange process is regulated by several classes of regulators, including GDP/GTP exchange factors (GEFs) and GTPase-activating proteins (GAPs) (David et al., 2012). But RND3 (also known as RhoE), a unique member of the Rnd subfamily, is found that it do not hydrolyze GTP, and thus remains constitutively active upon expression without regulation by GEFs/GAPs cycling (Jie et al., 2015).

Foster et al. (1996) firstly cloned RND3 which was shown to display no detectable GTPase activity, similar to RND1 and RND2, indicating that RND3 has a different manner in which to regulate its activity. Data from this study imply that RND3 may be a special small GTPase. The basic role of RND3 is to inhibit signaling downstream of RhoA to cause actin cytoskeleton dynamics, which contributes to migration, invasion of rounded tumor cells and neuron polarity. Besides, RND3 plays multiple roles in arresting cell cycle, inhibiting cell growth, and inducing apoptosis and differentiation (Belgiovine et al., 2010; Lin et al., 2013; Liu et al., 2016). Interestingly, previous research shows that RND proteins have an important function in the human uterus. It has been demonstrated that RND1, RND2, and RND3 proteins are expressed in the human myometrium, and the level of RND2 and RND3 proteins is upregulated during pregnancy (Lartey et al., 2006; Fortier et al., 2008; Lartey and Lopez Bernal, 2009). Moreover, Collett et al. (2012) found that RND3 promotes fusion of BeWo choriocarcinoma cells via cyclic AMP.

Forkhead box D3 (FOXD3) which is presented in human chromosome 1p31 is an important member of the FOX transcription factor family. It is originally shown that FOXD3 binds to DNA by identified with the consensus sequence 5′-A [AT]T[AG]TTTGTTT-3′ to initiate transcription (Sutton et al., 1996). FOXD3 functions in cell development is established as it has reported to be expressed in embryonic stem cells in the late-stage gastrula inner cell mass, in mouse and human embryonic stem cells, and is required in the preimplantation mouse embryo (Hanna et al., 2002; Tompers et al., 2005; Perera et al., 2006). FOXD3 as a tumor suppressor has been reported to affect growth, metastasis, invasion, and angiogenesis of many tumors. FOXD3 silencing in lung cancer cells was shown to promote cell growth and inhibit apoptosis (Yan et al., 2015; Xu et al., 2019).

Together, with evidence of RND3 associated with human diseases and abnormal animal phenotypes, these studies led us to examine its important role in the pathogenesis of diseases. Here, we found that RND3 expression was upregulated in patients with RM. To investigate expression and functional role of RND3 in trophoblasts, a series of experiments were performed in this study. We found that FOXD3 directly binds to the promoter regions of RND3 and enhances its protein expression. Besides, FOXD3 regulated RND3 function, including cell proliferation, apoptosis, and migration. Moreover, we further identified two pathways that regulated RND3 function.

## Materials and Methods

### Human Tissue Samples

Twenty-three patients with RM (age, 26–36 years) and twenty-four healthy pregnant women (HC) (age, 22–38 years) who had been treated at the Department of Obstetrics and Gynecology of the International Peace Maternity and Child Health Hospital of the China Welfare Institute, Shanghai Jiao Tong University School of Medicine, China, between April 2016 and March 2017. Patients were recruited did not have the following features: (1) chromosomal abnormality of parents or embryo, (2) abnormal immune function, (3) endocrine disorders, (4) abnormal uterine anatomy, (5) infectious disease, and (6) other identified causes of miscarriage.

The HC group have had previous pregnancies without spontaneous abortion, preterm labor, or PE and undergone painless induced abortions to terminate their unwanted pregnancies. Unwanted pregnancies refer to unintended pregnancies or unplanned pregnancies due to contraceptive failure. We exclude the patients who are unable to bear pregnancy because of physical condition, or whose fetus was diagnosed as malformation or congenital disease. Pregnancy interruption follows the individual will of women and is legal in China according to the *Chinese Maternal and Child Health Law*, *Women’s Rights Protection Law* and the *Population and Family Planning Law*. All villous tissue samples collected at 8–12 weeks of gestation were immediately collected, cleaned the clot with saline, and stored in liquid nitrogen. Two groups patients’ demographic information is showed in Table 1.

**TABLE 1:** Demographic information of the study population.

	Number	Age	Number of	Gestational
		(years)	miscarriages	age (weeks)
**HC**	24	30.08 ± 1.22	0.71 ± 0.16	10.74 ± 0.24
		(22–38)		
**RM**	23	30.65 ± 0.58	2.65 ± 0.29	10.30 ± 0.41
		(26–36)		
		*P* = 0.6807	*P* < 0.0001	*P* = 0.3661

The Medical Ethics Committee of International Peace Maternity and Child Health Hospital of the China Welfare Institute approved this study. Written informed consents were obtained from all patients who participated in the study before enrollment.

### Quantitative Real-Time PCR (qRT-PCR)

Total RNA was extracted from the villous tissue using TRIzol reagent (Life Technologies, Grand Island, NY, United States), and used to generate cDNA with the PrimeScript^TM^ RT reagent Kit with gDNA Eraser (RR047Q, Takara Bio, Kusatsu, Shiga, Japan). SYBR^®^ Premix Ex Taq (RR420A, Takara Bio) was used to perform PCR according to the manufacture’s instructions, on an ABI 7900 real-time PCR instrument. The PCR products were quantified using the 2^–ΔΔ*Ct*^ method relative to Glyceraldehyde-3-phosphate dehydrogenase (GAPDH) to normalized gene expression levels. The specific primers used are showed in Supplementary Table S1.

### Western Blot Analysis

Cells or tissue were lysed and analyzed by western blotting as described previously (Ma et al., 2017). Briefly, cells were washed twice with cold phosphate buffered saline (PBS) and harvested. Cell were lysed in radio immunoprecipitation assay buffer containing protease inhibitor on ice for 20 min. Proteins were detected using 10 or 12% polyacrylamide gels and transferred onto polyvinylidene fluoride (PVDF) membranes. 5% non-fat milk were used to block with PVDF membranes. Then they were incubated with primary antibody in 5% non-fat milk at 4°C overnight. The primary antibodies used are listed in Supplementary Table S2. After washing three times, membranes were incubated with secondary antibodies (1:5000; Yeasen, Shanghai, China) labeled with horseradish peroxidase (HRP). Signals were detected using an autoradiography film.

### Immunohistochemical and Immunofluorescence Staining of Tissues

Immunohistochemical staining was performed as described in our previous work (Li et al., 2019), using the Mouse- and Rabbit-specific HRP/DAB (ABC) Detection IHC Kit (ab64264; Abcam, Cambridge, United Kingdom) following the manufacturer’s protocol. Briefly, the tissue sections were deparaffinized and rehydrated. Epitope retrieval was performed in ethylenediaminetetraacetic acid (EDTA). After incubation with primary antibody overnight, HRP conjugated secondary antibody was used. For immunohistochemical detection, tissue was subsequently counterstained with diaminobenzidine, hematoxylin and hydrated. It is replaced the primary antibody with PBS as negative controls. Staining intensity was evaluated by ImageJ-Pro Plus 6.0 software. Pictures were captured under a Leica DMi8 microscope (Wetzlar, Germany).

Immunofluorescence staining of tissues was performed as described previously (Zhang et al., 2018).

### Cell Culture

The HTR-8/SVneo cell line (HTR-8, human extravillous trophoblast cell line, EVTs) were a kind gift from Dr. PK Lala (University of Western Ontario, ON, Canada). The cells were grown in Dulbecco’s modified Eagle’s medium (DMEM)/F12 plus 10% fetal bovine serum (FBS, Gibco, Grand Island, NY, United States) at 37°C with 5% CO_2_. Cells were cultured in a 10 cm^2^ dish, with a medium change every 48 h. For passaging, trypsin (Sigma-Aldrich, St. Louis, MO, United States) were used to detach cells at 37°C for 3 min.

### Small Interfering RNA (siRNA), Plasmids, and Transfection

RND3 and FOXD3 ON-TARGET plus SMART pool siRNAs and non-targeting siRNAs (siNC) were purchased from Thermo Scientific (Dharmacon RNAi Technologies, Lafayette, CO, United States; RND3: L-007794-00-0005, FOXD3: L-009152-00-0005, siNC: D-001810-10-15). HTR-8 cells were then transfected with 25 nM siRNA using DharmaFECT^TM^ Transfection reagents (Dharmacon RNAi Technologies) according to the manufacturer’s instructions. siROCK1-1 (5′-CCAGCUGCAAGCUAUAUUATT-3′) and siROCK1-2 (5′-GCAGAUGAAACAGGAAAUATT-3′) are purchased from GenePharma (Shanghai, China) and transfected into the cells at a final concentration of 100 nmol/L using oligofectamine reagent (Life Technologies). The RND3 overexpression plasmid was constructed by cloned the coding region sequence (CDS) of human RND3 into vector GV358 (GeneChem, Shanghai, China). JetPRIME^®^ reagent (Polyplus-transfection^®^ SA, Strasbourg, France) was used to perform cell transfection.

### Cell Proliferation Assay

After transfection about two thousand HTR-8 cells per well were plated in 96-well plates. Ten microliter per well the cell counting kit-8 (CCK-8) regents (Dojindo, Kumamoto, Japan) were added to cells to assess cell viability at 0, 24, 48, 72, and 96 h. After incubation with CCK-8 reagent for 2 h, the optical density value was detected at 450 nm using the Synergy H1 microplate reader (BioTek, Winooski, VT, United States).

### Immunofluorescence Staining of Cells

A suitable size slide was placed into 24-well plate each. HTR-8 cells cultured in a 24-well plate were fixed with 4% formaldehyde. Blocking buffer contained 0.3% Triton X-100 and 5% FBS was used to permeabilize cells for a minimum of 30 min. Then, cells were incubated with primary antibodies overnight (Supplementary Table S2). The next day, after washing three times with PBS cells were incubated with the appropriate secondary antibodies (1:1000; Thermo Fisher Scientific, Waltham, MA, United States). Concurrently, it is replaced the primary antibody with PBS alone as negative controls. HTR-8 cells were incubated for 2 h in the dark and washed with PBS. 4′,6-diamidino-2-phenylindole (DAPI; Abcam) was used to stain with the nuclei of HTR-8 cells. Slips which covered with cells were placed on the marked glass slides and images were captured under a microscope.

### Flow Cytometry Analysis for Apoptosis and Cell Cycle Assay

After transfection with siRNA or overexpression plasmid for 48 h, cells were detached and apoptosis was detected with the APC Annexin V Apoptosis Detection kit with 7-aminoactinomycin D (7-AAD) (BioLegend, Inc., San Diego, CA, United States) according to the manufacturer’s manual. For cell cycle analysis, cells were evaluated using propidium oxide using the Cycle Test Plus DNA Reagent kit (BD Biosciences, Franklin Lakes, NJ, United States) following the instruction manual. The percentage of apoptosis was analyzed by FlowJo software. And the percentage of cell cycle stage was analyzed by ModFit software. The apoptosis rate of HTR-8 cells in different phase was assessed.

### Transwell Assay

Transwell chambers (8 mm pores; Costar Corp., Cambridge, MA, United States) were used to perform cell migration assay as described previously (Tian et al., 2015). Briefly, serum-starved HTR-8 cells were detached and suspended. We loaded the upper chamber with 10^5^ cells in 200 μL 1% FBS DMEM/F12 medium and the chamber below with 700 μL medium with 15% FBS. After 24 h, non-migratory cells were removed gently and the migratory cells on the undersurface were fixed with 4% paraformaldehyde for 15 min and washed three times with PBS. 0.1% crystal violet was used to stain HTR-8 cells for 20 min and lastly we counted in 3–4 random fields to analyze.

### Explant Culture

Twenty-four-well culture dishes precoated with Matrigel^®^ substrate (Corning Life Sciences, New York, NY, United States) (Zhang et al., 2017) is prepared before one hour. Then first-trimester human placental villi (6–10 weeks) were immediately obtained from healthy patients after curettage surgery and put in cold ice during transport. And then the villi were dissected in small sections and explanted in overnight. About 8–10 tissue sections were placed into a well of a 24-well culture dish (Costar Corp.). The next day, villi that successfully grown in the Matrigel matrix were used for subsequent experiments, and were referred to as 24 h samples. Twenty-five nanometer siRND3 or 1 μg RND3 overexpression plasmid and their negative controls were transfected into the extravillous explants. Pictures were captured using a Leica microscope after 72 h overgrowth. ImageJ-Pro Plus 6.0 software was used to analysis cell migration.

### RND3 Promoter Luciferase Construction

A purification kit (Qiagen, Dusseldorf, Germany) was used to extract genomic DNA from HTR-8 cells and then the genomic DNA was used as a template for PCR. The RND3 promoter (nucleotides −1000 to +1, relative to the translation initiation site; GenBank accession number: MN685775, Supplementary Table S3) was synthesized by PCR and cloned into the plasmid pGL3-basic (Promega, Madison, WI, United States) with containing firefly luciferase activity by *Kpn*I and *Xho*I cleaved sites (Thermo Fisher Scientific). Following *E. coli* transformation, shaking, plasmid extraction, digestion, and purification steps were performed to successfully generate the RND3 promoter luciferase construct. Lastly, the right digestion fragment was sent to Sangon for sequencing (Shanghai, China). RND3 promoter luciferase report plasmid carrying mutations in the putative FOXD3 binding sites were generated using a QuikChange Lightning Muti Site-Directed Mutagenesis Kit (Stratagene, La Jolla, CA, United States).

For HTR-8 cells, the full-length FOXD3 cDNA was obtained from total RNA using PCR. The FOXD3 primers used were as follows: 5′-ATTGGATCCATGA CCCTCTCCGGCGGCG-3′ (forward) and 5′-AGGCTCGAGC TATTGCGCCGGCCATTTGG-3′ (reverse). The FOXD3 products were cloned into the vector pcDNA3.1 (+) (Invitrogen) to generate pcDNA3.1-FOXD3 overexpression plasmid.

### Dual-Luciferase Reporter Assay

HTR-8 cells were transfected with the control vector or FOXD3 overexpression plasmid, the RND3 promoter luciferase reporter plasmids and the Renilla luciferase expression vector pRL-TK (Promega), and using jetPRIME^®^ reagent. At 48 h post transfection, cells were lysed, and detected intracellular luciferase activity with a Dual-Luciferase^®^ Reporter Assay System (E1910; Promega) according to the manufacturer’s manual. Luciferase activities were detected with a microplate reader.

### Chromatin Immunoprecipitation (ChIP) Assay

The EZ-Magna ChIP^TM^A/G Kit (17-10086; Millipore, Billerica, MA, United States) was used to detect the binding of FOXD3 to RND3 according to the manufacturer’s manual. Five million cells were fixed in 1% formaldehyde for 10 min at room temperature. Cells were broken open and the crosslinked DNA was sheared to an average fragment size of 200–2000 bp. Subsequently, FOXD3 antibody (1 μg/10^5^ cells; Abcam) was used to immunoprecipitate with chromatin. The purified chromatin was subjected to qualitative PCR analysis using Premix Ex Taq^TM^ Hot Start Version (RR030Q; Takara) and quantified by qRT-PCR using SYBR^®^ Premix Ex Mix (Takara). The primer pairs used are showed in Supplementary Table S4. In this text, an input sample, 1% of starting chromatin was obtained from the ChIP assay each as a control for DNA contamination according the manufacturer’s protocol.

### Evaluation of the Activity of ROCK1

The ROCK1 activity was evaluated by immunoprecipitating ROCK1 and then performing an *in vitro* kinase activity assay (Mong and Wang, 2009; Han et al., 2016). Cells were lysed using a lysis buffer and then incubated for 1 h at 4°C with 2 μg of isotype control IgG, rabbit anti-ROCK1. And then the mixture was incubated with protein A/G agarose (Sigma-Aldrich) at 4°C overnight. Next day, the beads were pelleted, washed and incubated for 30 min at 30°C with a buffer containing 50 μM ATP and 0.5 μg of purified MYPT-1 (Millipore). Examination of phosphorylated MYPT-1 or immunoprecipitated ROCK1 was performed using western blotting.

### Statistical Analysis

Data are shown as means ± standard deviation (SD). All *in vitro* experiments were performed at least three times. Statistical analysis was calculated using the SPSS 15.1 (SPSS Inc., Chicago, IL, United States) and GraphPad Prism 5 (GraphPad Software, San Diego, CA, United States). Data were evaluated using Student’s *t*-test and two-way analysis of variance (ANOVA). A difference at *P* < 0.05 was considered statistically significant.

## Results

### RND3 Is Upregulated in Trophoblasts From Patients With RM

**FIGURE 1:**
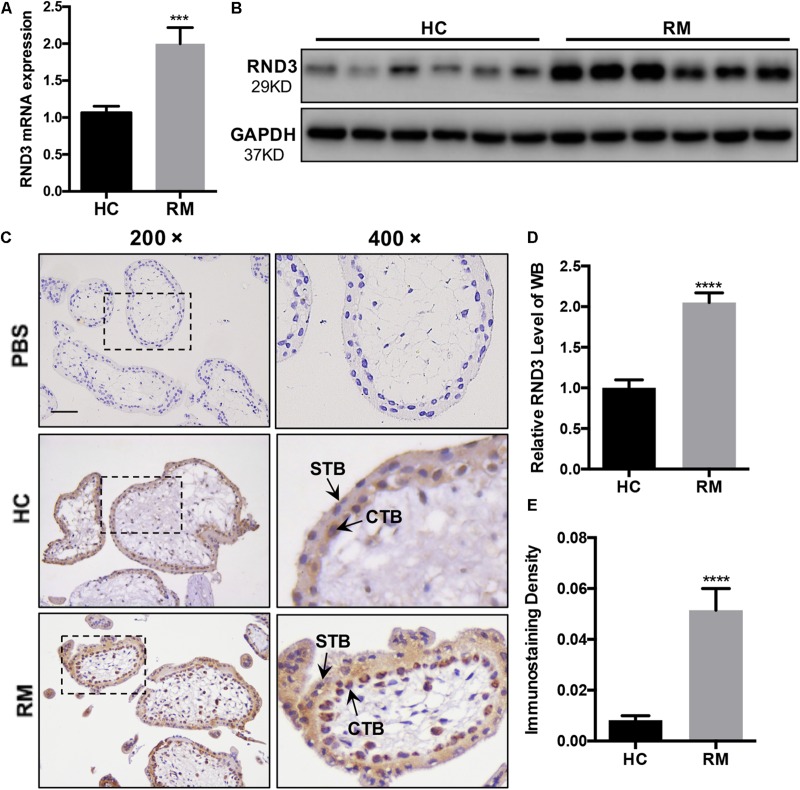
RND3 is upregulated in first-trimester placental CTB in patients with RM. **(A,B,D)** RND3 expression in first-trimester human villi tissues from patients with RM or HC was determined using qRT-PCR (*n* = 11) and western blot analysis (*n* = 6). **(C,E)** Immunohistochemical analysis of paraffin-embedded villous tissues showed that RND3 expression was increased in patients with RM compared with that in HC (*n* = 18). Scale bar = 100 μm. **(D)** Histogram showing the relative expression level of RND3 protein in HTR-8 cells as determined using ImageJ software. **(E)** The staining intensity of RND3 in both CTB and STB in paraffin-embedded villous tissues was quantified using Image-Pro Plus 6.0. CTB, cytotrophoblast; STB, syncytiotrophoblast. ***P < 0.001, ****P < 0.0001 vs. HC.

We first analyzed the RND3 expression at the first-trimester villi in patients with RM. qRT-PCR and western blotting results indicated that RND3 expressions were significantly increased in patients with RM as compared to those in HC (Figures 1A,B,D and Supplementary Figure S1). Immunohistochemistry results confirmed these findings (Figures 1C,E). We also found that the RND3 protein is present in the cytoplasm of CTB and syncytiotrophoblast (STB). According to previous studies, villous CTB cells as progenitor cells with a significantly proliferative ability differentiates into EVT or STB. Further, RND3 has been shown to promote the fusion of BeWo choriocarcinoma cells (Collett et al., 2012). In this text, we mainly explored the function of RND3 in CTB.

### RND3 Inhibits Trophoblast Proliferation, Blocks G1 Phase Cell Cycle Progression and Promotes HTR-8 Cell Apoptosis

**FIGURE 2:**
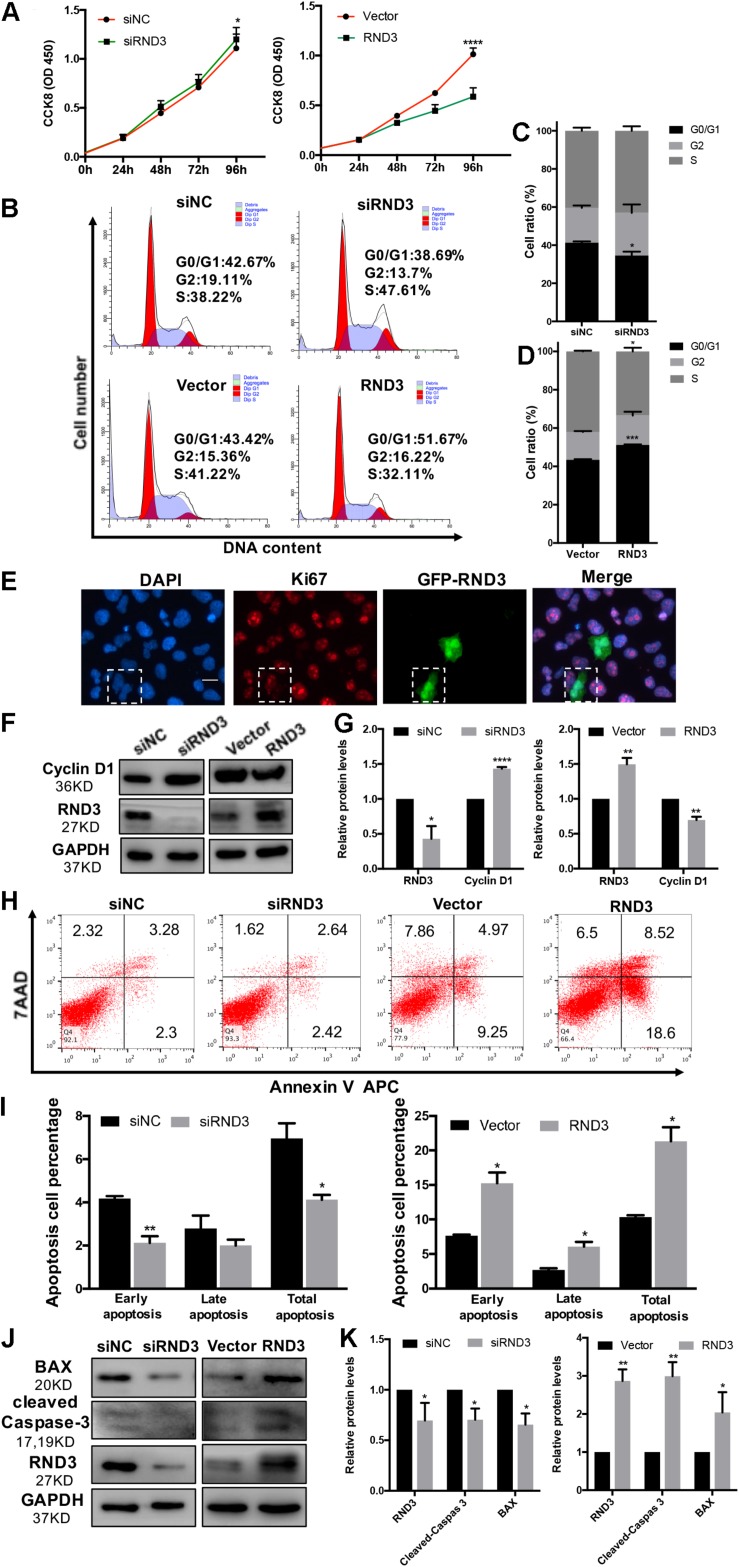
RND3 inhibits trophoblast proliferation, blocks G1 phase cell cycle progression and promotes HTR-8 cell apoptosis. **(A)** CCK-8 cell proliferation assay of RND3 knockdown and RND3-overexpressing HTR-8 cells and their respective control groups. **(B–D)** Cell cycle assay of RND3 knockdown and RND3-overexpressing HTR-8 cells and their respective control groups. The proliferation rate of cells in each phase was assessed. **(E)** Immunofluorescence staining with GFP-RND3 (green), Ki-67 (red), and DAPI (blue). Original magnification: ×200. Scale bar = 25 μm. **(F–G)** Western blot analysis of cyclin D1 levels in RND3 knockdown and RND3-overexpressing HTR-8 cells and their respective control groups. **(H,I)** Flow cytometry analysis of the apoptosis rate of RND3 knockdown and RND3-overexpressing HTR8 cells and their respective control groups. Histogram of Annexin V APC^+^/7-AAD^–^ represents early apoptotic cells. Histogram of Annexin V APC^+^/7-AAD^+^ represents late apoptotic cells. The sum of the former two categories equals the total number of apoptotic cells. **(J,K)** Western blot analysis of BAX and cleaved caspase-3 expression in RND3 knockdown and RND3-overexpressing HTR8 cells and their respective control groups. Data represent the means ± SD of three independent experiments. *P < 0.05, **P < 0.01, ***P < 0.001 vs. siNC or Vector group.

In our previous study, we demonstrated that the expression of Ki-67 expressed among proliferating cells was significantly decreased in patients with RM compared to those in HC (Li et al., 2019). Here, HTR-8 cells were transfected with siRND3 or RND3 overexpression construct to explore the function of RND3 (Supplementary Table S5). The CCK-8 assay revealed that RND3 knockdown promoted proliferation, whereas RND3 overexpression had the opposite effect (Figure 2A). In order to investigate how RND3 inhibits cell proliferation, we performed cell cycle assay. We found that downregulation of RND3 led to reduction in degrees of cells in the G0/G1phase (41.26 ± 0.706 vs. 34.55 ± 2.071) and upregulation of RND3 led to accumulation of cells in the G0/G1 phase (43.41 ± 0.332 vs. 51.23 ± 0.2569). And upregulation of RND3 led to reduction in degrees of cells in S phase (50.5 ± 1.637 vs. 37.32 ± 3.705). There were no differences in the percentage of cells in the G2/M phase (Figures 2B–D). Ki-67 which is only expressed in the proliferative phases such as G2/M and S phases was decreased after transfection with GFP-RND3 overexpression plasmid which transfection efficiency is around 50% by using GFP as a report gene (Figure 2E). The expression of cyclin D1, a G1-S phase cell cyclic protein, was increased in RND3 knockdown cells and decreased in RND3-overexpressing cells compared with that in their respective control groups (Figures 2F,G). Taken together, these experimental results suggest that RND3 inhibits cell proliferation by preventing S-phase entry.

In our previous study, TUNEL staining results showed accumulated apoptosis in the placenta villi from patients with RM (Wu et al., 2017). In this text, we further investigated the effect of RND3 on apoptosis by flow cytometry after Annexin V and 7-AAD staining. Our results showed that RND3 overexpression aggravated cell apoptosis at all stages, but RND3 knockdown attenuated early stage and total apoptosis (Figures 2H,I). The expression of BAX and cleaved caspase-3, two key components for cellular induced apoptosis, was increased in RND3-overexpressing cells and decreased in RND3 knockdown cells compared with that in their respective control groups (Figures 2J,K).

### RND3 Inhibits Trophoblast Migration *in vitro* and Outgrowth in a Villous Explant Culture Model

**FIGURE 3:**
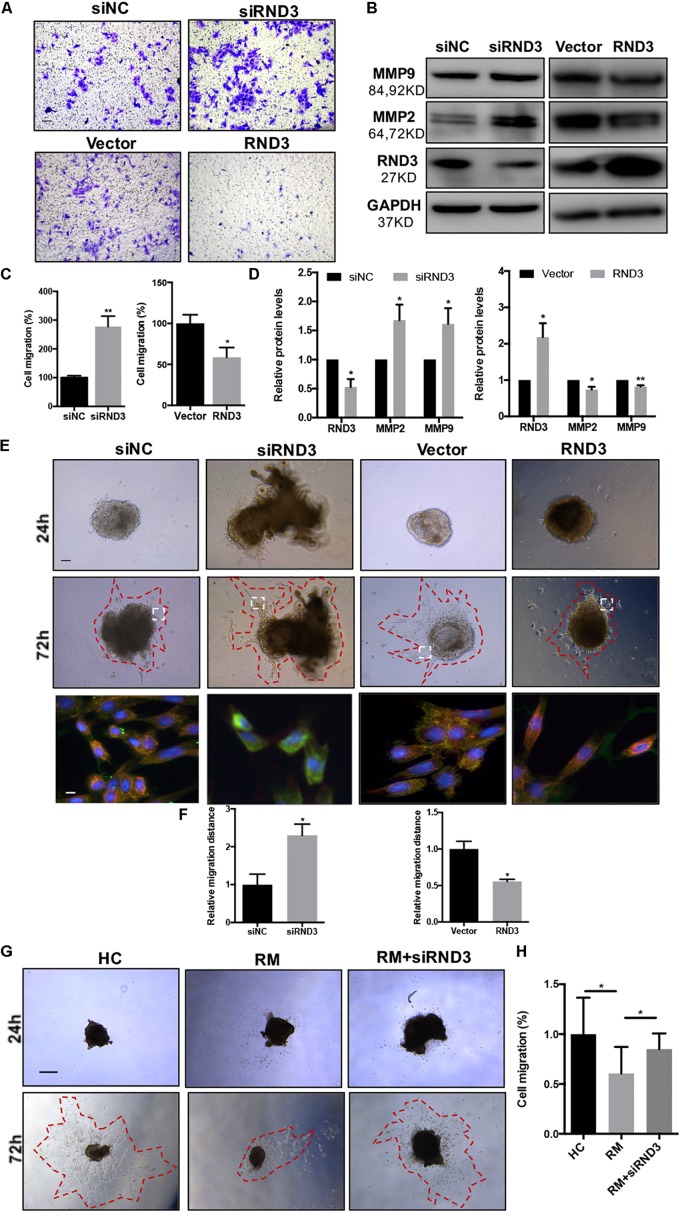
RND3 promotes trophoblast migration *in vitro*. **(A,C)** Transwell migration assay of RND3 knockdown and RND3-overexpressing HTR-8 cells and their respective control groups. Original magnification: ×100. Scale bar = 50 μm. **(B,D)** Western blot analysis of MMP-2 and MMP-9 expression in HTR-8 cells at 48 h after transfection with siNC, siRND3, control vector, or RND3 overexpression vector. **(E,F)** Villous explants were obtained from HC at 8–10 weeks of gestation and cultured on Matrigel. Tissues were then transfected with siRND3 or RND3 overexpression vector. Images were acquired after *in vitro* culture for 24 and 72 h. Original magnification: ×100. Scale bar = 100 μm. Immunofluorescence staining images of trophoblasts expressing RND3 (red) and CK7 (green). Original magnification: ×200. Scale bar = 25 μm. **(G,H)** Villous explants were obtained from HC and RM at 8–12 weeks of gestation and cultured on Matrigel. RM tissues were then transfected with siRND3. Images were acquired after *in vitro* culture for 24 and 72 h. Original magnification: ×40. Scale bar = 200 μm. Data represent the means ± SD of three independent experiments. *P < 0.05, **P < 0.01 vs. siNC, Vector, HC or RM group.

The migration and invasion potential of trophoblasts play an important role in embryo implantation. Hence, we explored the effect of RND3 on trophoblast migration. Transwell assay results showed that RND3 knockdown increased migration ability, while RND3 overexpression drastically decreased migration ability of HTR-8 cells (Figures 3A,C). Matrix metalloproteinase (MMP)-2 and MMP-9 are two vital members of the MMP family that can facilitate trophoblast cell movement by degrading fibronectin. MMP-2 and MMP-9 expression were found to be increased in RND3 knockdown cells and decreased in RND3-overexpressing cells compared with those in their respective control groups (Figures 3B,D). The function of RND3 in trophoblast migration *in vitro* was verified by villous explants derived from healthy samples cultured on Matrigel-coated plates. Immunofluorescence staining and western blotting confirmed the effects of knocking down or overexpressing RND3 using cytokeratin 7 (CK7) as trophoblast cell marker to identify trophoblast in the villous plants (Supplementary Figure S2). Results showed that RND3 knockdown or RND3-overexpressing enhanced or weakened the migration ability of trophoblasts, respectively (Figures 3E,F). To further confirm the role of RND3 in trophoblast invasion and migration *in vivo*, explants were obtained from HC and RM and planted into Matrigel. After 24 h of culture, the explants from RM anchored on to the Matrigel were separated into two groups. They were treated with siNC and siRND3. At 72 h of *in vitro* culture, RM group explants showed smaller migration and proliferation capacities than HC group. However, the hindered migration of RM group can be rescued by siRND3 treatment (Figures 3G,H). All these results demonstrated that RND3 plays an importance role in trophoblast migration.

### Verification of RND3 Core Promoter Region and FOXD3 as a Key Transcriptional Enhancer for RND3

**FIGURE 4:**
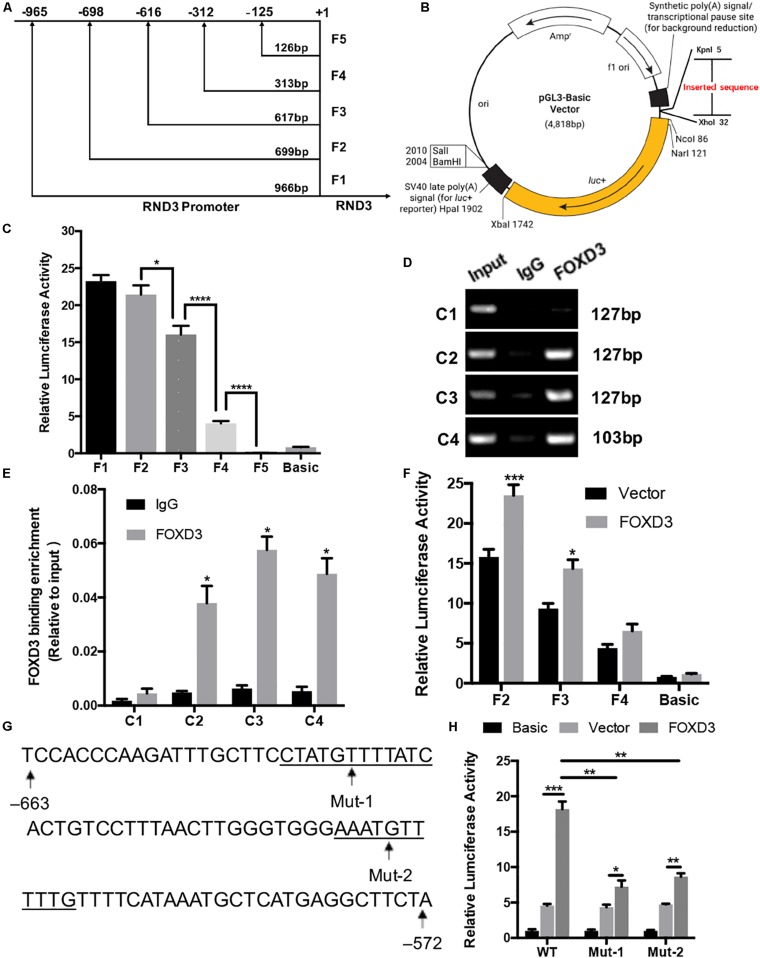
Identification of RND3 core promoter region and FOXD3 as a transcriptional enhancer for RND3 in HTR-8 cells. **(A)** Specific primers for sequences upstream of the RND3 transcription initiation site were designed and **(B)** cloned into pGL3-basic vector. **(C)** Relative luciferase activity of F1–F5 and basic plasmids. *P < 0.05, ****P < 0.0001. **(D,E)** ChIP assay of the combination of RND3 promoter. *P < 0.05 vs. the IgG group. **(F)** Relative luciferase activity of F2–F4 and basic plasmids in HTR-8 cells at 48 h after transfection with control vector or RND3 overexpression vector. *P < 0.05 vs. Vector group. **(G)** Prediction of two FOXD3 binding sites in the RND3 core promoter region (–663 to –572) (underlined) and construction of the FOXD3 binding site mutant reporters. **(H)** Relative luciferase activity of RND3 reporter plasmids (WT) and FOXD3 binding site mutant reporters (Mut-1 and Mut-2). Data represent the means ± SD of three independent experiments. *P < 0.05, **P < 0.01, ***P < 0.001 vs. Vector group or FOXD3 group.

We selected a 1000 bp region (positive −1000 to +1) located upstream of the RND3 initiation codon as the core promoter region. According to the forecasting result, we designed the different length fragments (966, 699, 617, 313, and 126 bp) of RND3 promoter. These fragments were subjected to PCR amplification using total DNA from human trophoblasts (Supplementary Table S6). After identifying a series of incomplete promoter fragments by double-enzyme digestion, they were subcloned into the pGL3-basic luciferase reporter vector. The positive samples were subjected to sequencing, and the results were as expected, indicating that the vectors were successfully constructed and named F1–F5 (Figures 4A,B). HTR-8 cells were transfected with the reporter plasmids and pRL-TK plasmids. After 48 h, reporter activity was measured by dual-luciferase reporter assay. Results showed that the activity of F4 plasmid was significantly higher than that of F5 plasmid or basic plasmid, while that of F3 plasmid was significantly increased. The activity of F2 plasmid was higher than that of F3 plasmid (Figure 4C). These results indicated the presence of important regulatory elements at the promoter region of −125 to −698 bp.

Next, we attempted to identify the transcription factor at −125 to −698 bp region that regulates RND3 expression using the TRANSFAC tool (ALGGEN-PROMO and JASPAR). According to the candidate transcription factor score, relative score, dissimilarity, matching degree, and some other standards, we identified FOXD3 as the highest scoring transcription factor in the −125 to −698 bp region (Supplementary Tables S7, S8). We focused on the sequence with a candidate transcription factor score greater than 6.5, a relative score greater than 0.8, relatively smaller dissimilarity and positive strand. At the same time, we removed some repetitive sequences in one method and chose the same segments in two tools. To determine binds to this genomic locus, ChIP assay was performed in HTR-8 cells. Four pairs of RND3 primers were designed for standard end-point PCR and qRT-PCR using the DNA obtained from the ChIP assay. ChIP results indicated that FOXD3 binds directly to the identified promoter region of RND3 (−649 to −239 bp) (Figures 4D,E). The reporter plasmids F2–F4 were transfected into HTR-8 cells in conjunction with the control plasmid or FOXD3 expression plasmid. Dual-luciferase reporter assay showed that the activity of F2 and F3 plasmids increased after transfection with FOXD3 overexpression plasmid compared with that after transfection with control vector (Figure 4F). Based on the above results, we predicted two FOXD3 binding sites in the RND3 core promoter region (−663 to −572). Furthermore, two luciferase reporters were constructed with control of either the wild-type RND3 reporter plasmid (WT, also F1 plasmids) or two mutants in which two putative FOXD3 binding sites had been mutated (Mut-1 and Mut-2 reporter, respectively) (Figure 4G). As expected, the activity of the reporter plasmid was decreased in HTR-8 cells which were transfected with Mut-1 or Mut-2 reporter as compared with transfection in WT reporter (Figure 4H). These results suggest that FOXD3 acts as a transcriptional enhancer for RND3 in HTR-8 cells.

### FOXD3 Is Upregulated in Trophoblasts From Patients With RM

**FIGURE 5:**
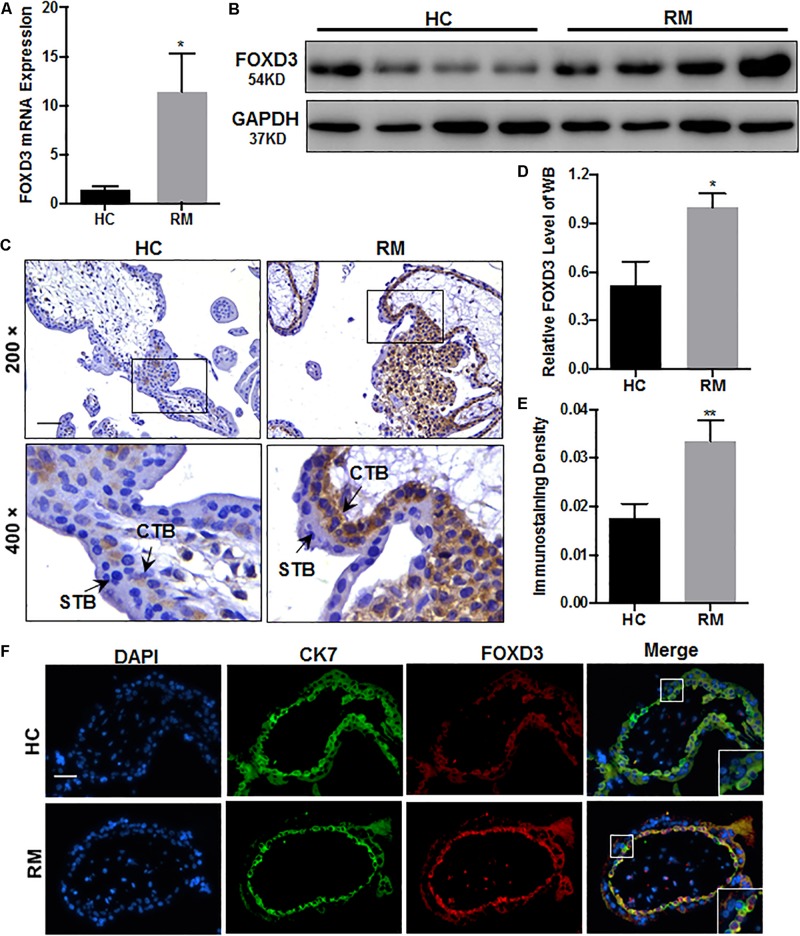
FOXD3 is upregulated in first-trimester placental CTB in patients with RM. **(A,B,D)** FOXD3 expression in first-trimester human villi tissues from patients with RM or HC was determined using qRT-PCR (*n* = 10) and western blot analysis (*n* = 6). **(C,E)** Immunohistochemical analyses of paraffin-embedded villous tissue showed that FOXD3 expression was increased in patients with RM compared with that in HC (*n* = 17). Original magnification: ×200. Scale bar = 100 μm. **(D)** Histogram showing the relative expression level of FOXD3 protein in HTR-8 cells as determined using ImageJ software. **(E)** The staining intensity of FOXD3 in paraffin-embedded villous tissues was quantified using Image-Pro Plus 6.0. **(F)** Two-color immunofluorescence staining analyses of paraffin-embedded villous tissues revealed expression of FOXD3 (red) and CK7 (green), and counterstaining with DAPI (blue). Original magnification: ×200. Scale bar = 50 μm. CTB, cytotrophoblast; STB, syncytiotrophoblast. *P < 0.05, **P < 0.01 vs. HC.

Next, we explored the FOXD3 level at the first-trimester villi in patients with RM. qRT-PCR and western blotting results indicated that FOXD3 expression was increased in patients with RM as compared to those in HC (Figures 5A,B,D). Immunohistochemical and immunofluorescence staining results further confirmed these findings (Figures 5C,E,F). Our results also demonstrated that the FOXD3 protein was present in the cytoplasm of CTB, with almost no expression in STB.

### FOXD3 Regulates RND3 Expression, Inhibits Proliferation and Migration, and Promotes Apoptosis in HTR-8 Cells

**FIGURE 6:**
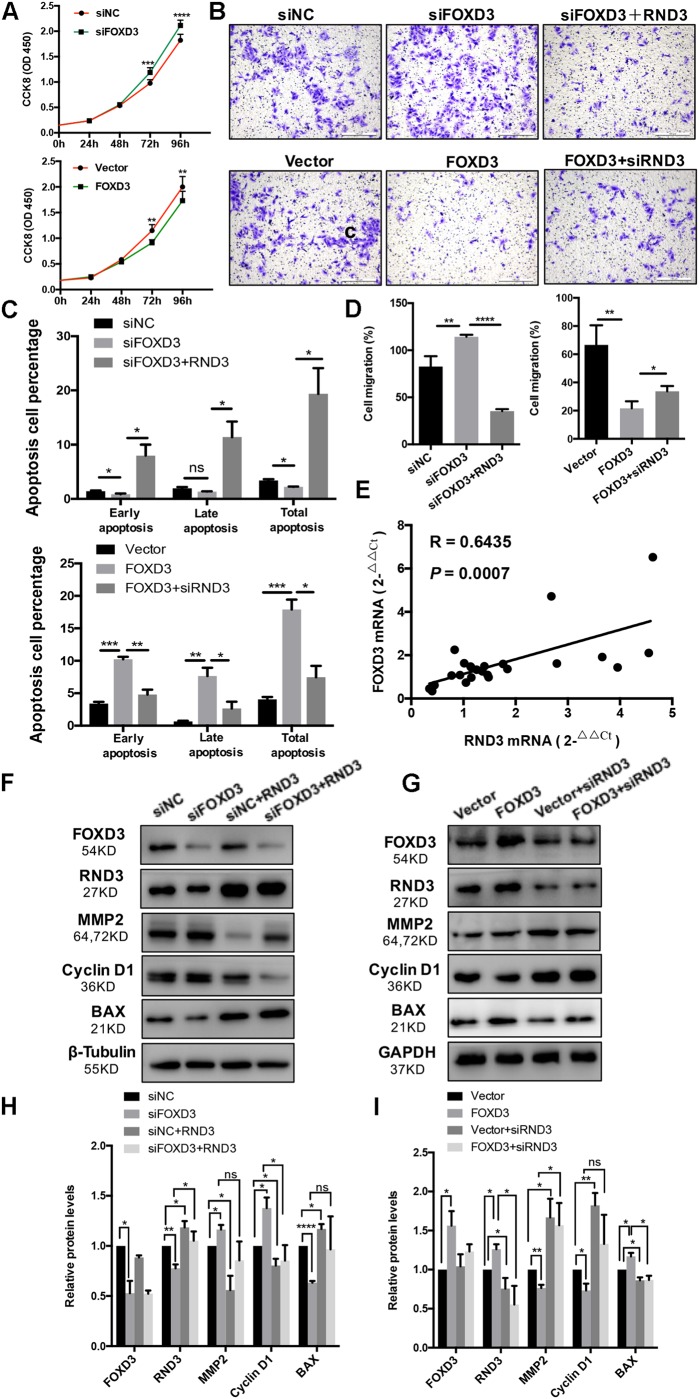
FOXD3 regulates RND3 expression, inhibits proliferation and migration, and promotes apoptosis in HTR-8 cells *in vitro*. **(A)** CCK-8 assay of FOXD3 knockdown and FOXD3-overexpressing HTR8 cells and their respective control groups. **(B,D)** Transwell migration assay of FOXD3 knockdown without or with RND3 overexpression and FOXD3-overexpressing without or with siRND3 HTR-8 cells and their respective control groups. Original magnification: × 100. Scale bar = 100 μm. **(C)** Flow cytometry analysis of the apoptosis rate of siNC, siFOXD3, siFOXD3 + RND3 and Vector, FOXD3, FOXD3 + siRND3 group. Histogram of Annexin V APC^+^/7-AAD^–^ represents early apoptotic cells. Histogram of Annexin VAPC^+^/7-AAD^+^ cells represents late apoptotic cells. The sum of the former two categories equals the total number of apoptotic cells. **(E)** The mRNA expression level of RND3 and FOXD3 in the villous tissues of patients with RM and HC was determined using qRT-PCR (*n* = 24). RND3 mRNA expression was correlated with FOXD3 mRNA expression in villous tissues. Correlation analysis was performed using Spearman’s rank correlation test. **(F–I)** Western blot analysis of FOXD3, RND3, MMP2, Cyclin D1, and BAX expression in HTR-8 cells at 48 h after transfection with siNC and siFOXD3 or control vector and FOXD3 overexpression vector. AT 24 h after transfection with siFOXD3 or FOXD3 overexpression vector, HTR-8 cells were transfected RND3 overexpression vector or siRND3 again. Data represent the means ± SD of three independent experiments. *P < 0.05, **P < 0.01, ***P < 0.001, ****P < 0.0001 vs. siNC, Vector, siFOXD3 or FOXD3 group.

As FOXD3 directly regulates RND3 expression, we examined whether FOXD3 influences the biological behavior of trophoblasts. The results of CCK-8 assay showed that knockdown of FOXD3 promoted HTR-8 cell proliferation, while overexpression of FOXD3 inhibited HTR-8 cell proliferation (Figure 6A). In order to claim that FOXD3 regulates RND3 function, we transfected FOXD3-overexpressing cells with siRND3 or FOXD3 knockdown cells with RND3 overexpression plasmid, respectively. Transwell assay results showed that FOXD3 overexpression inhibited HTR-8 cell migration and RND3 knockdown can reverse the effect. In addition, FOXD3 knockdown-promoted HTR-8 migration was weakened by RND3 overexpression (Figures 6B,D). Flow cytometry analysis showed that FOXD3 knockdown decreased the ratio of early apoptotic and total apoptotic cells but did not alter the proportion of late apoptotic cells, while RND3 overexpression can reverse the effects. FOXD3 overexpression increased the ratio of early apoptotic and late apoptotic cells, and RND3 knockdown can suppress the effect (Figure 6C). RND3 and FOXD3 mRNA expression in first-trimester villi in patients with RM and HC was analyzed by qRT-PCR. Results showed that RND3 mRNA expression was correlated positively with FOXD3 mRNA expression in the first-trimester villi (Figure 6E). Western blot analysis showed that Cyclin D1, MMP2 level was upregulated and BAX level was downregulated in HTR-8 cells transfected with siFOXD3 as compared with that in their respective control groups (Figures 6F,H). In contrast, Cyclin D1, MMP2 level was downregulated and BAX level was upregulated in cells transfected with FOXD3 overexpression plasmid (Figures 6G,I). In addition, RND3 overexpression or RND3 knockdown can partly rescue the change in FOXD3 knockdown cells or in FOXD3-overexpressing cells (Figures 6F–I). These results confirmed that FOXD3 acts as a transcriptional enhancer for RND3 in HTR-8 cells and regulates RND3 function.

### RND3 Regulates Trophoblast Migration and Proliferation via the RhoA- Rho-Associated Coiled-Coil Containing Kinase 1 (ROCK1) Signaling Pathway

**FIGURE 7:**
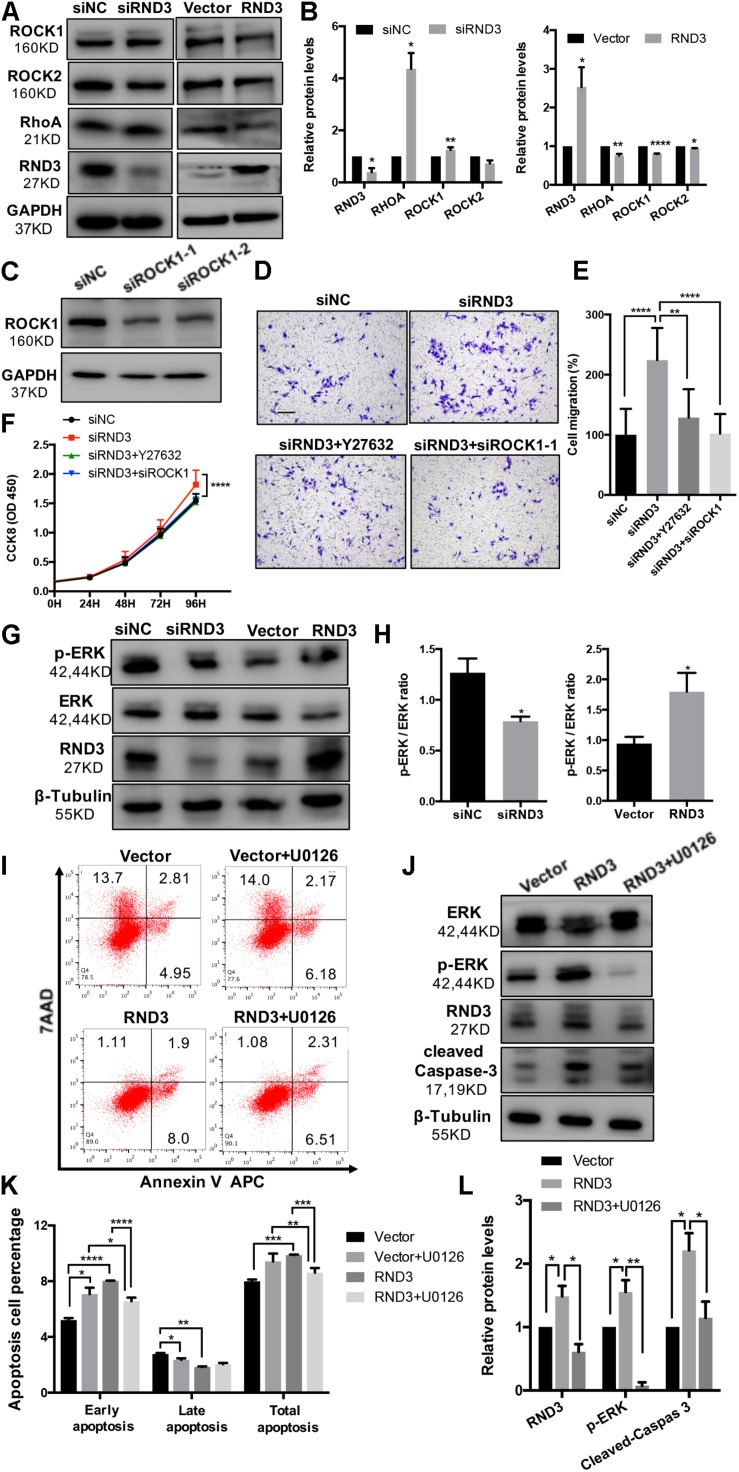
RND3 regulates trophoblast proliferation and migration via the RhoA-ROCK1 signaling pathway and regulates trophoblast apoptosis via the ERK1/2 signaling pathway. **(A,B)** Western blot analysis of ROCK1, ROCK2, and RhoA expression in HTR-8 cells at 48 h after transfection with siNC and siRND3 or control vector and RND3 overexpression vector. **(C)** Western blot showing ROCK1 levels in HTR-8 cells transfected with siROCK1. **(D,E)** HTR-8 cells were transfected with siNC, siRND3, siROCK1 for 36 h, and then treated with Y-27632 for 12 h. Trophoblast migration was assessed by crystal violet staining. Original magnification: ×100. Scale bar = 100 μm. **(F)** CCK-8 assay of siNC- and siRND3-transfected HTR-8 cells in the presence or absence of Y-27632 or siROCK1. **(G,H)** Western blot analysis of p-ERK and ERK expression in HTR-8 cells at 48 h after transfection with siNC and siRND3 or control vector and RND3 overexpression vector. **(I,K)** HTR-8 cells were transfected with control vector or RND3 overexpression vector for 36 h, and then treated with U0126 for 12 h. The apoptosis rate was assessed by flow cytometry. **(J,L)** Western blot analysis of p-ERK, ERK, cleaved caspase-3, and RND3 expression in HTR-8 cells at 48 h after transfection with control vector or RND3 overexpression vector in the presence or absence of U0126. Data represent the means ± SD of three independent experiments. *P < 0.05, **P < 0.01, ***P < 0.001, ****P < 0.0001 vs. siNC, Vector or RND3 group.

Previous findings have suggested that RND3 is closely related to the RhoA-ROCK pathway (Riento et al., 2005; Riento and Ridley, 2006). Hence, we analyzed ROCK1/2 and RhoA expression in HTR-8 cells after RND3 interference. Results showed that ROCK1 and RhoA expression was increased in RND3 knockdown cells and decreased in RND3-overexpressing cells (Figures 7A,B). However, no significant difference in ROCK2 expression between both these groups was observed. Besides, ROCK1 activity was examined by measuring the phosphorylation state of a major ROCK1 substrate, MYPT-1, at Thr853 by western blotting. We found that p-MYPT1 was upregulated in RND3 knockdown cells consistent with the change of total ROCK1 level (Supplementary Figure S3C). siROCK1 and Y-27632 (Selleck Chemicals), a specific inhibitor of ROCK, was used to inhibit ROCK expression. Western blot analysis revealed that Y-27632 can inhibit ROCK1 expression at concentrations ranging from 5 to 25 μM. Hence, we chose 10 μM as a working concentration for following experiments (Supplementary Figure S3A). CCK-8 and transwell assay results showed that Y-27632 and siROCK1 treatment inhibited proliferation and migration in RND3 knockdown HTR-8 cells (Figures 7C–F). However, flow cytometry results showed no differences in migration and proliferation in the presence or absence of Y-27632 or siROCK1 in RND3 knockdown HTR-8 cells (Supplementary Figure S4). These findings indicate that RND3 regulates trophoblast migration and proliferation via the RhoA-ROCK1 signaling pathway, but ROCK1 is not involved in RND3-induced apoptosis in HTR-8 cells.

### RND3 Regulates Trophoblast Apoptosis via the Extracellular Signal-Regulated Protein Kinases 1 and 2 (ERK1/2) Signaling Pathway

In order to identify the unknown pathways or key molecules involved in trophoblast apoptosis, we explored various signaling pathways, including the p53, phosphatidylinositol-3 kinase-protein kinase B, JAK (Janus kinase)/STAT, and ERK1/2 pathways. We discovered that the expression of phosphorylated ERK1/2 (p-ERK) was decreased in RND3 knockdown HTR-8 cells and increased in RND3-overexpressing cells (Figures 7G,H). In order to determine the part of the ERK1/2 pathway in RND3 function, U0126 (Sigma-Aldrich), an inhibitor of mitogen-activated protein kinase (MAPK), was used to inhibit the ERK1/2 pathway. Western blot analysis revealed that U0126 can inhibit phosphorylated ERK1/2 expression at concentrations ranging from 5 to 25 μM. Hence, we chose 5 μM as a working concentration for following experiments (Supplementary Figure S3B). Flow cytometry results showed that although treatment with U0126 increased the percentage of apoptotic cells, U0126 can rescue some percentage of apoptosis caused by overexpression-RND3 in HTR-8 cells (Figures 7I,K). Besides, western blotting results revealed that cleaved caspase-3 expression was decreased in RND3-overexpressing HTR-8 cells in the presence U0126 compared with that in the absence of U0126 (Figures 7J,L). Interesting, we found that p-ERK level in first-trimester human villi tissues from patients with RM or HC was upregulated by western blot analysis (Supplementary Figures S5A,B). Immunohistochemical analyses of paraffin-embedded villous tissue showed that p-ERK protein is present in cytoplasm of CTB (Supplementary Figure S5C). These results suggest that RND3 regulates HTR-8 cell apoptosis via the ERK1/2 pathway.

In order to further understand the regulatory mechanism of RND3, we explored the relation between the RhoA/ROCK1 and ERK1/2 pathways. Interestingly, western blot analysis showed that Y-27632 treatment increased p-ERK expression and U0126 treatment increased ROCK1 expression (Supplementary Figure S6). These findings indicate that ROCK1 and ERK1/2 act as antagonists in HTR-8 cells.

## Discussion

An in depth understanding of the molecular mechanisms and functions of RND3 in different tissues is required to identify novel therapeutic targets for the diagnosis and treatment of human diseases (Jie et al., 2015). RND3 plays a complex role in cells. It can regulate cell proliferation and plays a key role in regulating metastasis and invasion in tumor cells (Paysan et al., 2016). Accumulating evidence has shown that RND3 plays an important role in the regulation of trophoblast function (Collett et al., 2012; Xu et al., 2017; Li et al., 2018); however, there are no studies reporting its function in RM and little is known about its underlying regulatory mechanism in trophoblasts.

**FIGURE 8:**
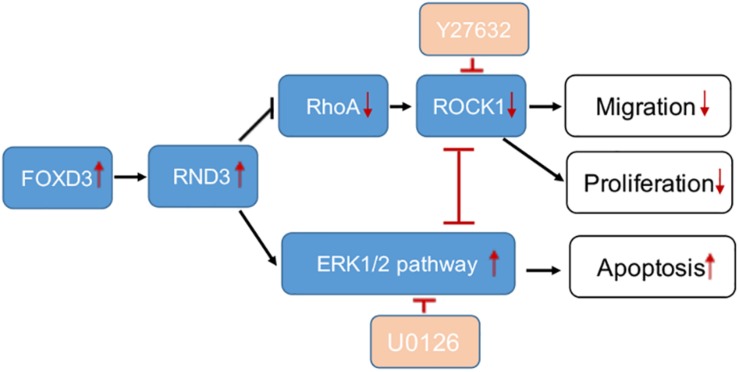
A schematic illustration of the role of RND3 in trophoblast function. RND3 regulates trophoblast migration, proliferation, and apoptosis through different but related pathways. The red arrows show the changes in patients with RM compared with HC. The red T sign indicates the inhibitor effect.

Our study provides the first insight into the function of RND3 in CTB proliferation, apoptosis, and migration and its potential character in RM (Figure 8). Our results showed that RND3 was expressed in CTB and STB in first-trimester human placental villous tissues, and its expression in patients with RM was significantly higher than that in normal villi (Figure 1). Further, we found that overexpression of RND3 suppressed HTR-8 cell proliferation and blocked G1 phase cell cycle progression. In addition, overexpression of RND3 promoted HTR-8 cell apoptosis (Figure 2). These data are consistent with the results that trophoblasts of RM exhibit a low percentage of proliferation and a high percentage of apoptosis. Normal CTB function play a vital role for embryo implantation, spiral artery remodeling and maternal-fetal communication. Abnormal CTB proliferation and apoptosis show a connection with RM and many other pregnancy-associated disease, such as PE and fetal growth restriction (Wu et al., 2016). Besides, our study demonstrated that overexpression of RND3 suppressed HTR-8 cell migration (Figure 3). RM is related with the dysfunction of proliferative and migratory capacities of trophoblasts. Thus, RND3-targeted strategies may provide a new choice for the diagnosis and treatment of RM patients with trophoblasts dysfunction in early pregnancy.

We systemically investigated the transcriptional regulation of RND3 in HTR-8 cells using ChIP and dual-luciferase reporter assays. We found that FOXD3 functioned as a transcriptional enhancer for RND3 and predicted two FOXD3 binding sites in the RND3 core promoter region (Figure 4). So far, little is known about the function of FOXD3 compared to other members of the FOX family in adult cells. Many experimental evidences suggest that FOXD3 is a transcriptional repressor in adult cells. However, in our study, we found that FOXD3 directly binds to the promoter regions of RND3 to regulate RND3 function, including inhibition of proliferation and migration and promotion of apoptosis in HTR-8 cells (Figure 6), thus suggesting a novel mechanism underlying the regulatory effect of FOXD3 in RM. Moreover, our results demonstrated that FOXD3 and RND3 were highly expressed in the CTB of patients with RM and a positive correlation between their transcript levels was observed (Figure 6E).

Rho GTPases which can regulate cell migration, cell cycle and cell morphogenesis are regarded as vital downstream targets of G protein-coupled receptors (Sah et al., 2000). ROCK activity has been involved in the function of migration of tumor cells, particularly in amoeboid cell motility (Hu et al., 2019; Steurer et al., 2019). Many evidences suggested that RhoA and ROCK play important role in the migration of first-trimester EVT cells (Shiokawa et al., 2002). In our study, we demonstrated that RND3 suppressed RhoA-ROCK activates and expression and the pathway mediated HTR-8 cells migration and proliferation of RND3 (Figures 7A–F). Next, we found that the level of p-ERK was decreased in RND3 knockdown HTR-8 cells and increased in RND3-overexpressing cells (Figures 7G,H). ERK1/2, as members of the MAPK superfamily, mainly regulate cell proliferation and apoptosis. Some studies have suggested that the balance between the intensity and duration of pro-apoptotic or anti-apoptotic signals conveyed by ERK1/2 determines the proliferation or apoptosis of cells (Mebratu and Tesfaigzi, 2009). Sinha et al. (2004) found that withdrawal of soluble survival factors from mouse renal proximal tubular cells lead to apoptosis which was induced by ERK1/2 activity and U0126 or PD98059 can inhibit the activity. The stimuli type and the cell type which provide sufficient ERK1/2 substrates determine that ERK1/2-induced signaling lead to proliferation or apoptosis (Sinha et al., 2004). In the present study, we found that knockdown of RND3 increased ERK1/2 activation-induced apoptosis. However, inhibition of ERK1/2 phosphorylation in RND3-overexpressing HTR-8 cells by U0126 treatment decreased ERK1/2-induced apoptosis. Moreover, previous findings have indicated that ERK1/2 and ROCK1 correlate in a signaling network. Hensel et al. (2014) found that there was a two-way stream of information between ROCK and ERK, with inter-suppression of both molecules in NSC34 cells. But, this process became an unidirectional crosstalk, ultimately leading to dysregulation of neurite outgrowth in a model of spinal muscular atrophy (Hensel et al., 2014). Using western blot analysis, we demonstrated that ROCK and ERK were mutual inhibited in HTR-8 cells (Supplementary Figure S6). These data indicate that RND3 function is regulated by these two pathways (Figure 8).

## Conclusion

In conclusion, our study showed that RND3 and FOXD3 expression was significantly increased in the CTB of patients with RM. FOXD3 is a key transcription factor that binds to the RND3 core promoter region and regulates RND3 expression, inhibits proliferation and migration, and promotes apoptosis in HTR-8 cells. Besides, RND3 regulates CTB migration and proliferation via the RhoA-ROCK1 signaling pathway and inhibits apoptosis via the ERK1/2 pathway in HTR-8 cells. Our findings emphasize the importance of RND3 to human pregnancy and provide the basis for the development of molecular therapies for RM. Nevertheless, further research is required for the application of theory to clinical practice.

## Data Availability Statement

The nucleotide sequence has been deposited to GenBank, accession number: MN685775. Other raw data supporting the conclusion of this article will be made available by the authors, without undue reservation, to any qualified researcher.

## Ethics Statement

The studies involving human participants were reviewed and approved by the Medical Ethics Committee of International Peace Maternity and Child Health Hospital of the China Welfare Institute, Shanghai Jiao Tong University School of Medicine. The patients/participants provided their written informed consent to participate in this study.

## Author Contributions

X-LM and XL performed the experiments. X-LM and H-QM contributed to the statistical analyses. SQ and Y-CZ provided the material support. F-JT, W-HZ, L-QS, YZ, and YL provided funding support. JZ, YZ and YL contributed valuable advice to the development of the manuscript. X-LM and YL wrote the manuscript.

## Conflict of Interest

The authors declare that the research was conducted in the absence of any commercial or financial relationships that could be construed as a potential conflict of interest.
