# Supplementary material for: Upregulation of RND3 Affects Trophoblast Proliferation, Apoptosis, and Migration at the Maternal-Fetal Interface
Source: Front Cell Dev Biol. 2020 Mar 13;8:153. doi: 10.3389/fcell.2020.00153 (PMC7083256; doi:10.3389/fcell.2020.00153)
Supplement: Supplementary file 1 [file Table_1.docx]

**Supplementary Table 1.** Primers used for qRT-PCR

| **Primer** | **Primer sequence(5'-3')** |
| --- | --- |
| GAPDH-F | TGGAGTCCACTGGCGTCTTC |
| GAPDH-R | TGCTGATGATCTTGAGGCTGTTG |
| RND3-F | TTGAGCCTGTGGGACACTTC |
| RND3-R | AGATCAGACTTGCAGCCGAC |
| FOXD3-F | AAGCCCAAGAACAGCCTAGTG |
| FOXD3-R | TGACGAAGCAGTCGTTGAGT |
