# Supplementary material for: Upregulation of RND3 Affects Trophoblast Proliferation, Apoptosis, and Migration at the Maternal-Fetal Interface
Source: Front Cell Dev Biol. 2020 Mar 13;8:153. doi: 10.3389/fcell.2020.00153 (PMC7083256; doi:10.3389/fcell.2020.00153)
Supplement: Supplementary file 2 [file Table_2.docx]

**Supplementary Table 2. Antibodies used in this study.**

| Antibody | WB | IHC | IF | ChIP | IP | Supplier and catalogue number |
| --- | --- | --- | --- | --- | --- | --- |
| RND3 | 1μg/ml |  |  |  |  | R&D Systems (MAB6618) |
| RND3 |  | 1:100 |  |  |  | Novus (NBP2-49315) |
| RND3 |  |  | 1:100 |  |  | Santa Cruz Biotechnology (sc-53874) |
| GAPDH | 1:5000 | - | - |  |  | Abcam (ab181602) |
| Cyclin D1 | 1:2000 |  |  |  |  | Abcam (ab134175) |
| BAX | 1:1000 |  |  |  |  | CST (5023) |
| Cleaved caspase-3 | 1:1000 |  |  |  |  | CST (9661) |
| CK7 | - | - | 1:100 |  |  | CST (4465S) |
| MMP9 | 1:1000 | - | - |  |  | CST (13667S) |
| MMP2 | 1:1000 | - | - |  |  | Novus (NB200-114SS) |
| β-Tubulin | 1:1000 | - |  |  |  | CST (2128S) |
| FOXD3 | 1:250 | 1:200 | 1:100 |  |  | Santa Cruz Biotechnology (sc-517206) |
| FOXD3 |  | - | - | 1μg/10^5^cell |  | Abcam (ab67758) |
| ROCK1 | 2 μg/ml |  |  |  |  | R&D Systems (MAB4590) |
| ROCK1 |  |  |  |  | 2-5 μg | Santa Cruz Biotechnology(sc-17794) |
| ROCK2 | 1:500 |  |  |  |  | Santa Cruz Biotechnology (sc-365275) |
| RhoA | 1:1000 |  |  |  |  | CST (2117) |
| p-ERK | 1:1000 | 1:500 |  |  |  | CST (4370) |
| ERK | 1:1000 |  |  |  |  | CST (4695) |
| p-MYPT | 1:1000 |  |  |  |  | CST(4563) |
| MYPT | 1:1000 |  |  |  |  | CST(2634) |

Abbreviations: WB, western blotting; IHC, immunohistochemistry; IF, immunofluorescence; ChIP, chromatin immunoprecipitation.
