# Supplementary material for: Upregulation of RND3 Affects Trophoblast Proliferation, Apoptosis, and Migration at the Maternal-Fetal Interface
Source: Front Cell Dev Biol. 2020 Mar 13;8:153. doi: 10.3389/fcell.2020.00153 (PMC7083256; doi:10.3389/fcell.2020.00153)
Supplement: Supplementary file 3 [file Table_3.docx]

**Supplementary Table 3.** The promoter fragment of RND3.

ACCAGTCCCCTCACTGCAAATGTGGGGAGGTGCAACTAAGCTGACAAGTGTTTGGGTCCCTAGCGCAGTAGGGTTGAGTTTCCAATGGCCTTTGTCACTCAGTCATGGGCATCTTCCACTTCTCTTTCTTCTCCCCTCCCACCTCCTGTACCCACCCCTCGAAACCTTTCTGCCTCCACTTGTACGACTGAAACTGACAGGTTTCAGAAGCCAGTCTACTACCGTGACCCAGGCAAGCCTGTTCTTGCATCTGGAATAAAGAGGCTGATCTATTGGTCCATGGCCTATGTACCTGATACAAACCATTAAAAAAAAAAATAGAACCACTCAACTATTCTCCACCCAAGATTTGCTTCCTATGTTTTATCACTGTCCTTTAACTTGGGTGGGAAATGTTTTTGTTTTCATAAATGCTCATGAGGCTTCTACAGATAAGCCCCTATTAAACTATATTTAAAGGGATTGTAGTTCAAATCAGTATGCACAGATACCAGTTTCCCACCTTGCCCTGTGGACTTGTCAGCAAAGCATCTTTGGGATCTTCATGTTGTCTAAGAGCCTTGGTCTAAAATAGATCCCGAATGGGCACCCTAGAGGGCGCACTGCTTACATTTTCGGCATGCAACGCCAACAGCGCTCGCTCCCAGGCTCCATAAAAGGGGAGGAGGCAGATCAGTTTCCTCCTCTCATTGAGAAAGAAGAGGATTGGAAGGCCGGGCGTATAAAGCCGCGCAGAGAGCGTGAAACAAAGCAGTCGGCTCGGAATTGGACTTGGGAGGCGCGGTGAGGAGTCAGGCTTAAAACTTGTTGGAGGGGAGTAACCAGCCTGCTCCTCTCGCTCTCCTCCTCGTCTGCGCCGCGTTTCAGAGGTTGCCCATCAGCCTTGTGATTTATTTTTATATCTGCTTTTTATAAAGAGAGAAATATATATATATATATATATTTTTTTTTTTCTTCTTAAGAGAAAATTCCTGTTCCAAGAGAAAATAAGGCAACATCAATGAAGGAGAGAAGAGCCAGC

The underlined part is the promoter fragment of －1000 to +1. The yellow highlighted part indicates the intiation codon.The red highlighted nucleotide is intiation codon.
