# Supplementary material for: Upregulation of RND3 Affects Trophoblast Proliferation, Apoptosis, and Migration at the Maternal-Fetal Interface
Source: Front Cell Dev Biol. 2020 Mar 13;8:153. doi: 10.3389/fcell.2020.00153 (PMC7083256; doi:10.3389/fcell.2020.00153)
Supplement: Supplementary file 4 [file Table_4.docx]

**Supplementary Table 4.** Primers used in ChIP assay.

| **Primer** | **Primer sequence(5'-3')** | **Size/bp** | **Start to End site** |
| --- | --- | --- | --- |
| C1-F | GGCAAGCCTGTTCTTGCATC | 127 | -769 to -643 |
| C1-R | AGGAAGCAAATCTTGGGTGGA |  |  |
| C2-F | GCTTCCTATGTTTTATCACTG | 127 | -649 to -523 |
| C2-R | CTGATTTGAACTACAATCCC |  |  |
| C3-F | CCTTGCCCTGTGGACTTGT | 127 | -499 to -373 |
| C3-R | GCGTTGCATGCCGAAAATGT |  |  |
| C4-F | GGGAGGAGGCAGATCAGTTT | 103 | -341 to -239 |
| C4-R | CGAGCCGACTGCTTTGTTTC |  |  |
