# Supplementary material for: Upregulation of RND3 Affects Trophoblast Proliferation, Apoptosis, and Migration at the Maternal-Fetal Interface
Source: Front Cell Dev Biol. 2020 Mar 13;8:153. doi: 10.3389/fcell.2020.00153 (PMC7083256; doi:10.3389/fcell.2020.00153)
Supplement: Supplementary file 5 [file Table_5.docx]

**Supplementary Table 5.** The expression of RND3 in trophoblast cell lines HTR-8, JAR and Bewo.

| Cell lines | RND3(Ct) | GAPDH(Ct) | ΔCt | 2^-ΔΔCt^ |
| --- | --- | --- | --- | --- |
| **HTR-8** | 18.91±0.77 | 15.02±0.56 | 3.90±0.24 | 2019 |
| **JAR** | 24.44±1.03 | 14.80±0.47 | 9.64±1.43 | 38 |
| **Bewo** | 29.52±1.43 | 14.64±0.32 | 14.88±1.50 | 1 |
