# Supplementary material for: Upregulation of RND3 Affects Trophoblast Proliferation, Apoptosis, and Migration at the Maternal-Fetal Interface
Source: Front Cell Dev Biol. 2020 Mar 13;8:153. doi: 10.3389/fcell.2020.00153 (PMC7083256; doi:10.3389/fcell.2020.00153)
Supplement: Supplementary file 6 [file Table_6.docx]

**Supplementary Table 6.** Primer sequences for the different missing fragments of RND3 promoter

| **Primer** | **Primer sequence(5'-3')** | **Size/bp** |
| --- | --- | --- |
| F1 | CGGGGTACCCTAAGCTGACAAGTGTTTGG | 966 |
| F2 | CGGGGTACCCCATTAAAAAAAAAAATAGAACCAC | 699 |
| F3 | CGGGGTACCGGGAAATGTTTTTGTTTTCATAA | 617 |
| F4 | CGGGGTACCATTGAGAAAGAAGAGGATTGG | 313 |
| F5 | CGGGGTACCCATCAGCCTTGTGATTTATTTTTAT | 126 |
| R | CCG*CTCGAG*TTGATGTTGCCTTATTTTCT |  |

The underlined part is the Kpn I enzyme cutting site.

The underlined and italics part is the Xhol enzyme cutting site.
