# Supplementary material for: Upregulation of RND3 Affects Trophoblast Proliferation, Apoptosis, and Migration at the Maternal-Fetal Interface
Source: Front Cell Dev Biol. 2020 Mar 13;8:153. doi: 10.3389/fcell.2020.00153 (PMC7083256; doi:10.3389/fcell.2020.00153)
Supplement: Supplementary file 7 [file Table_7.docx]

**Supplementary Table 7.** PROMO TFBS prediction.

| **Factor name** | **Start position** | **End position** | **Dissimilarity** | **String** | **RE equally** | **RE query** |
| --- | --- | --- | --- | --- | --- | --- |
| FOXD3 [T02290] | 45 | 52 | 3.81788 | AAGTGTTT | 0.07637 | 0.09451 |
| FOXD3 [T02290] | 356 | 363 | 1.387772 | CTATGTTT | 0.09164 | 0.12656 |
| FOXD3 [T02290] | 390 | 397 | 0.461834 | AAATGTTT | 0.0611 | 0.09082 |
| FOXD3 [T02290] | 396 | 403 | 1.018305 | TTTTGTTT | 0.0611 | 0.09155 |
| FOXD3 [T02290] | 743 | 750 | 1.387772 | AAACAAAG | 0.09164 | 0.12656 |
| FOXD3 [T04166] | 307 | 317 | 13.371894 | AAAAAAAAAAA | 0.04009 | 0.07161 |
| FOXD3 [T04166] | 308 | 318 | 12.585312 | AAAAAAAAAAT | 0.01002 | 0.01948 |
| FOXD3 [T04166] | 394 | 404 | 14.158476 | GTTTTTGTTTT | 0.06014 | 0.0989 |
| FOXD3 [T04166] | 941 | 951 | 12.585312 | ATTTTTTTTTT | 0.01002 | 0.01948 |
| FOXD3 [T04166] | 942 | 952 | 13.371894 | TTTTTTTTTTT | 0.04009 | 0.07161 |
