# Supplementary material for: Upregulation of RND3 Affects Trophoblast Proliferation, Apoptosis, and Migration at the Maternal-Fetal Interface
Source: Front Cell Dev Biol. 2020 Mar 13;8:153. doi: 10.3389/fcell.2020.00153 (PMC7083256; doi:10.3389/fcell.2020.00153)
Supplement: Supplementary file 8 [file Table_8.docx]

**Supplementary Table 8.** JASPAR TFBS prediction.

| **Matrix ID** | **Name** | **Score** | **Relative score** | **Start** | **End** | **Strand** | **Predicted sequence** |
| --- | --- | --- | --- | --- | --- | --- | --- |
| MA0041.1 | Foxd3 | 12.4056 | 0.917065911 | 938 | 949 | + | ATATATTTTTTT |
| MA0041.1 | Foxd3 | 11.8426 | 0.905832647 | 391 | 402 | + | AAATGTTTTTGT |
| MA0041.1 | Foxd3 | 10.3307 | 0.875670039 | 309 | 320 | - | TATTTTTTTTTT |
| MA0041.1 | Foxd3 | 10.3307 | 0.875670039 | 941 | 952 | + | TATTTTTTTTTT |
| MA0041.1 | Foxd3 | 10.0684 | 0.87043838 | 940 | 951 | + | ATATTTTTTTTT |
| MA0041.1 | Foxd3 | 9.95163 | 0.868107883 | 308 | 319 | - | ATTTTTTTTTTT |
| MA0041.1 | Foxd3 | 9.95163 | 0.868107883 | 942 | 953 | + | ATTTTTTTTTTT |
| MA0041.1 | Foxd3 | 8.72343 | 0.843605154 | 357 | 368 | + | CTATGTTTTATC |
| MA0041.1 | Foxd3 | 8.63703 | 0.841881416 | 310 | 321 | - | CTATTTTTTTTT |
| MA0041.1 | Foxd3 | 8.61652 | 0.841472166 | 312 | 323 | - | TTCTATTTTTTT |
| MA0041.1 | Foxd3 | 8.46607 | 0.838470793 | 922 | 933 | - | ATATATATATTT |
| MA0041.1 | Foxd3 | 8.46607 | 0.838470793 | 934 | 945 | + | ATATATATATTT |
| MA0041.1 | Foxd3 | 8.34674 | 0.836090011 | 889 | 900 | + | ATTTATTTTTAT |
| MA0041.1 | Foxd3 | 8.26967 | 0.834552519 | 936 | 947 | + | ATATATATTTTT |
| MA0041.1 | Foxd3 | 7.98593 | 0.828891699 | 943 | 954 | + | TTTTTTTTTTTC |
| MA0041.1 | Foxd3 | 7.37152 | 0.816634141 | 945 | 956 | + | TTTTTTTTTCTT |
| MA0041.1 | Foxd3 | 7.14748 | 0.812164445 | 397 | 408 | + | TTTTGTTTTCAT |
| MA0041.1 | Foxd3 | 6.8892 | 0.807011696 | 393 | 404 | + | ATGTTTTTGTTT |
| MA0041.1 | Foxd3 | 6.70686 | 0.803374074 | 296 | 307 | - | AATGGTTTGTAT |
