# Supplementary material for: Upregulation of RND3 Affects Trophoblast Proliferation, Apoptosis, and Migration at the Maternal-Fetal Interface
Source: Front Cell Dev Biol. 2020 Mar 13;8:153. doi: 10.3389/fcell.2020.00153 (PMC7083256; doi:10.3389/fcell.2020.00153)
Supplement: Supplementary file 9 [file Table_9.docx]

**Supplementary Figure Legend**

**Supplementary Figure 1. The uncropped image of Western blot for RND3 in Fig 1b.**

**Supplementary Figure 2. Immunofluorescence staining and western blotting confirmed the effects of knocking down or overexpressing RND3.** (a) Representative immunofluorescence of RND3 in primary trophoblasts from first-trimester villous tissue of HC patients. Fluorescence signals specific to CK7 appear green, RND3 appear red, and the DAPI appear blue. Original magnification: ×100. Scale bars: 100 μm. (b) Western blotting verify the RND3 expression in explanted villi after siRND3 and RND3 plasmid transfection.

**Supplementary Figure 3. The appropriate concentration of Y-27632 and U0126 are chose.** (a) Western blotting results showed ROCK1 and RhoA expression increased following Y-27632 treatment. (b) Western blotting results showed that p-ERK and ERK expression increased following U0126 treatment. (c) ROCK1 activity was examined by measuring the p-MYPT-1 by western blotting in RND3 knockdown or RND3-overexpressing cells.

**Supplementary Figure 4.** **ROCK1 is not involved in RND3-induced apoptosis in HTR-8 cells.** (a) Flow cytometry analysis of the apoptosis rate of siNC- and siRND3-transfected HTR-8 cells in the presence or absence of Y-27632 or siROCK1. (b) Histogram of Annexin V APC+/7-AAD− represents early apoptotic cells. Histogram of Annexin V APC+/7-AAD+ represents late apoptotic cells. The sum of the former two categories equals the total number of apoptotic cells. Data represent the means ± SD of three independent experiments. **P* < 0.05, ***P* < 0.05 vs siNC group.

**Supplementary Figure 5. p-ERK is upregulated in first-trimester CTB in patients with RM.** (a, b) p-ERK expression in first-trimester human villi tissues from patients with RM or HC was determined using western blot analysis (*n*=7). (c) Immunohistochemical analyses of paraffin-embedded villous tissue showed that p-ERK protein is present in cytoplasm of CTB. Scale bar = 100 μm. **P* < 0.05 vs siNC group.

**Supplementary Figure 6. Mutual inhibition of ROCK1 and ERK1/2 pathways.** (a, c) Western blot analysis of ROCK1, RhoA, p-ERK, and ERK expression in HTR-8 cells treated with or without Y-27632 for 6 h. (b, d) Western blot analysis of ROCK1, p-ERK, and ERK expression in HTR-8 cells treated with or without U0126 for 3 h. Data represent the means ± SD of three independent experiments. **P* < 0.05, ***P* < 0.01 vs DMSO group.
