# Supplementary figures and images for: Upregulation of RND3 Affects Trophoblast Proliferation, Apoptosis, and Migration at the Maternal-Fetal Interface
Source: Front Cell Dev Biol. 2020 Mar 13;8:153. doi: 10.3389/fcell.2020.00153 (PMC7083256; doi:10.3389/fcell.2020.00153)

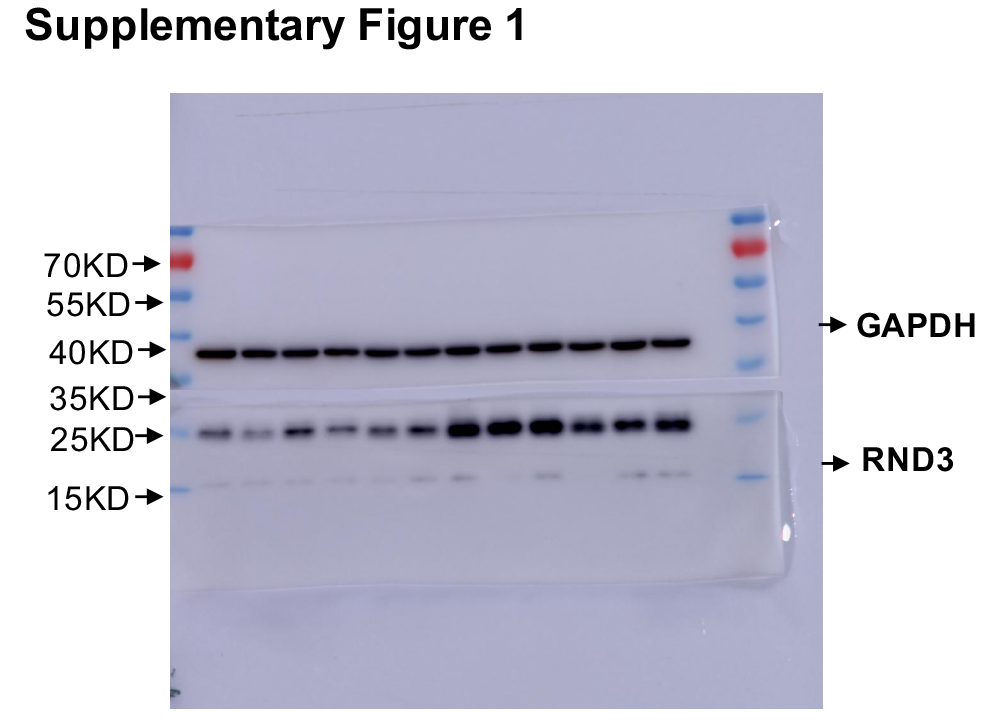

Supplement: Supplementary file 10 [file Image_1.tif]

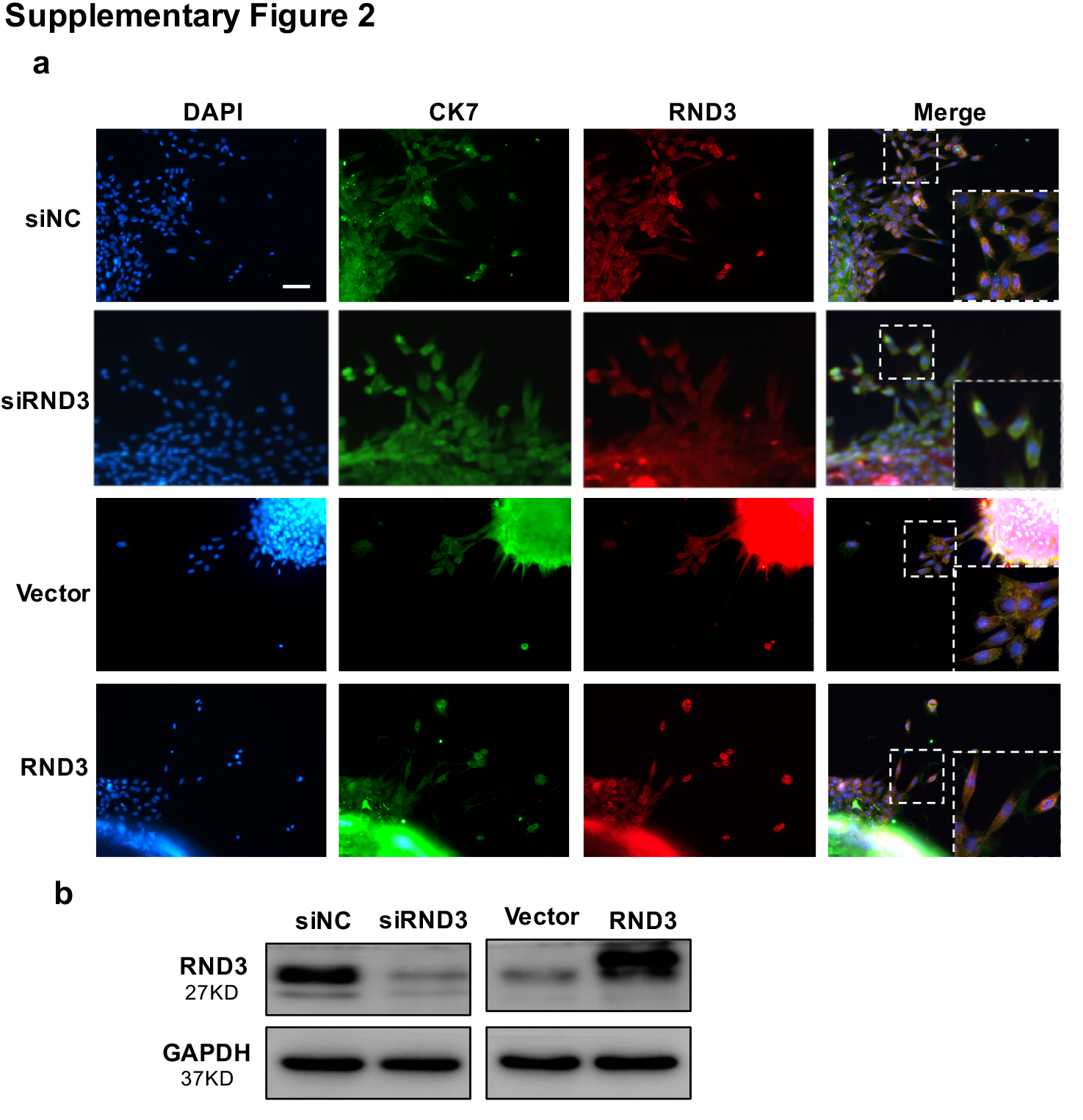

Supplement: Supplementary file 11 [file Image_2.tif]

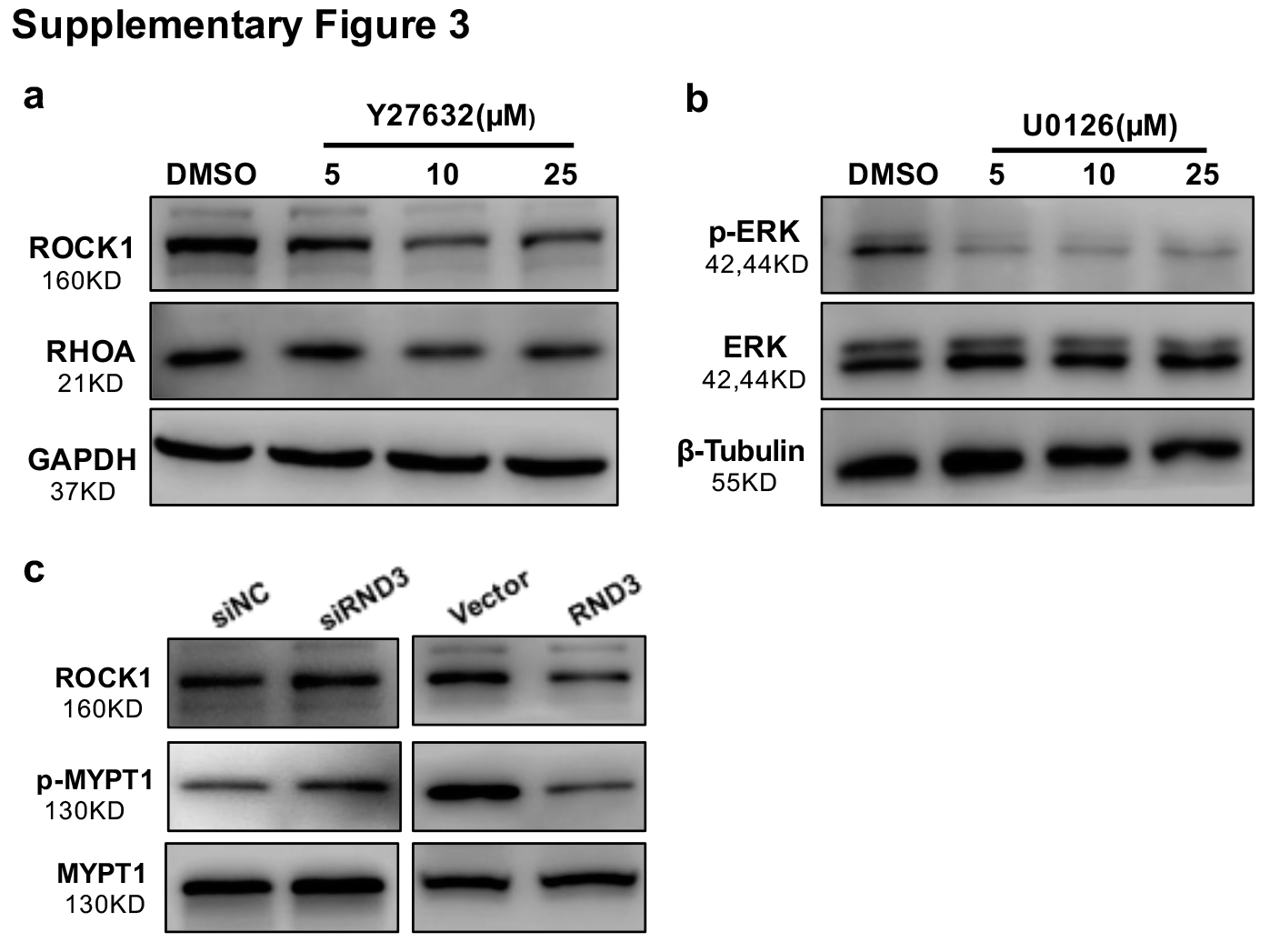

Supplement: Supplementary file 12 [file Image_3.tif]

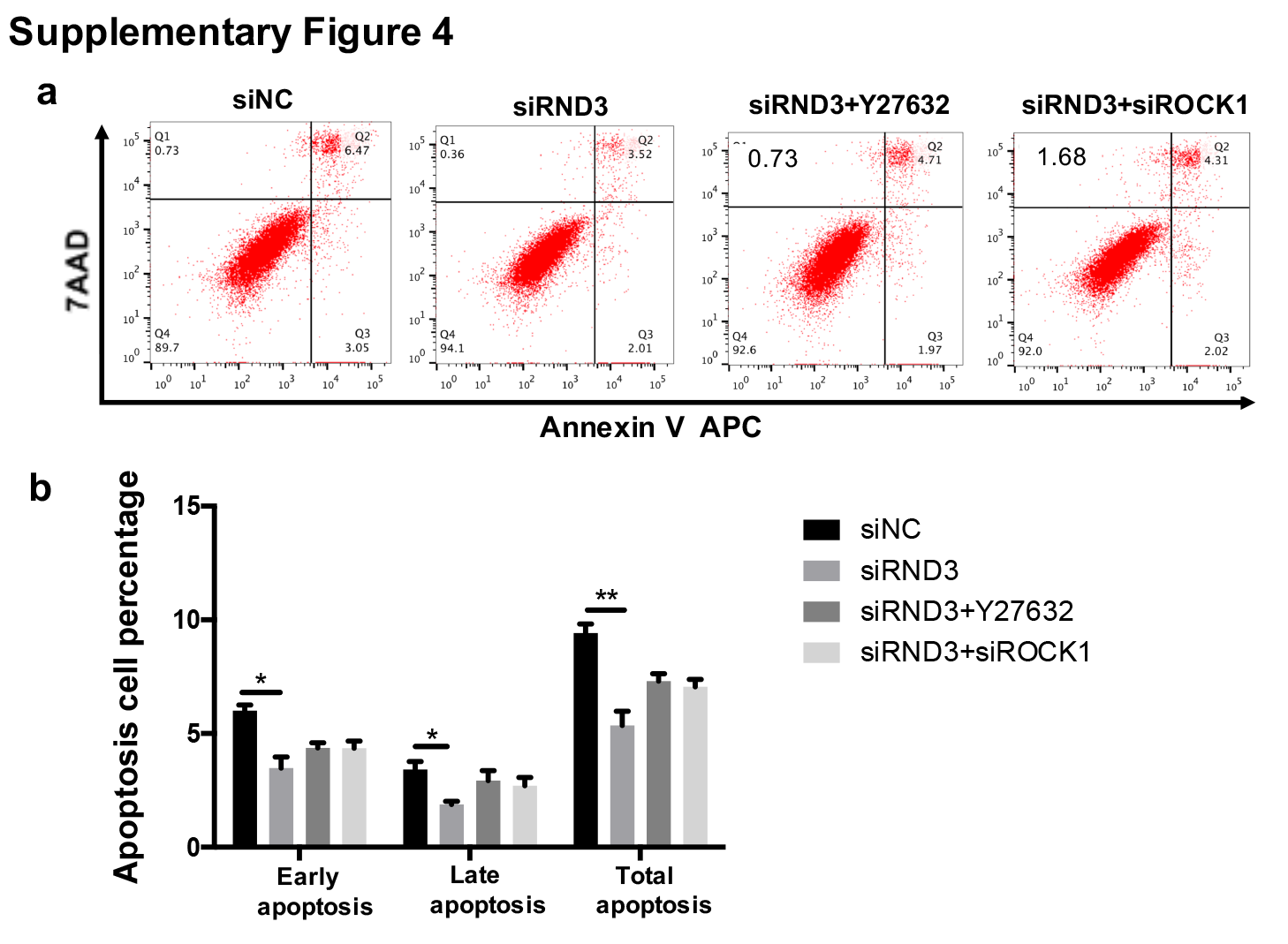

Supplement: Supplementary file 13 [file Image_4.tif]

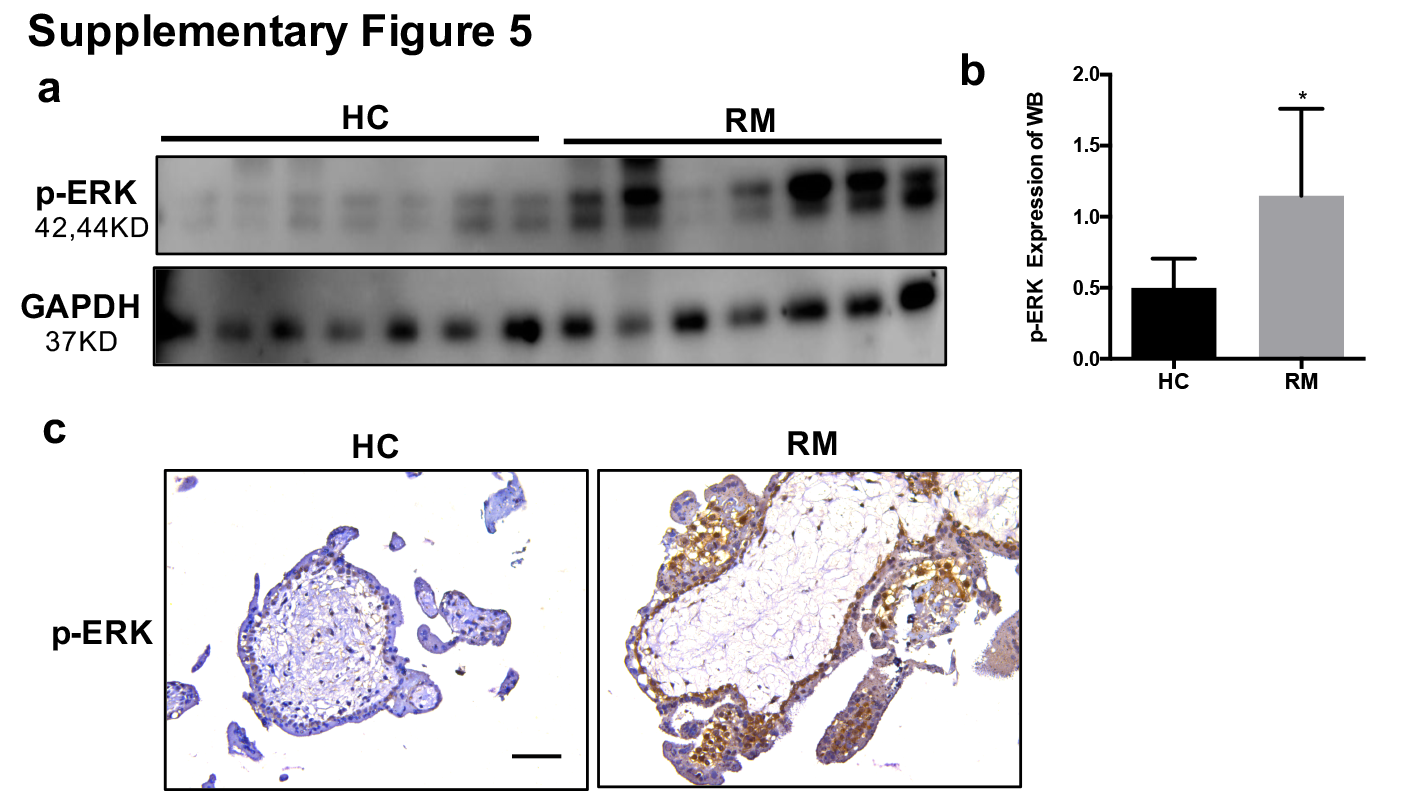

Supplement: Supplementary file 14 [file Image_5.tif]

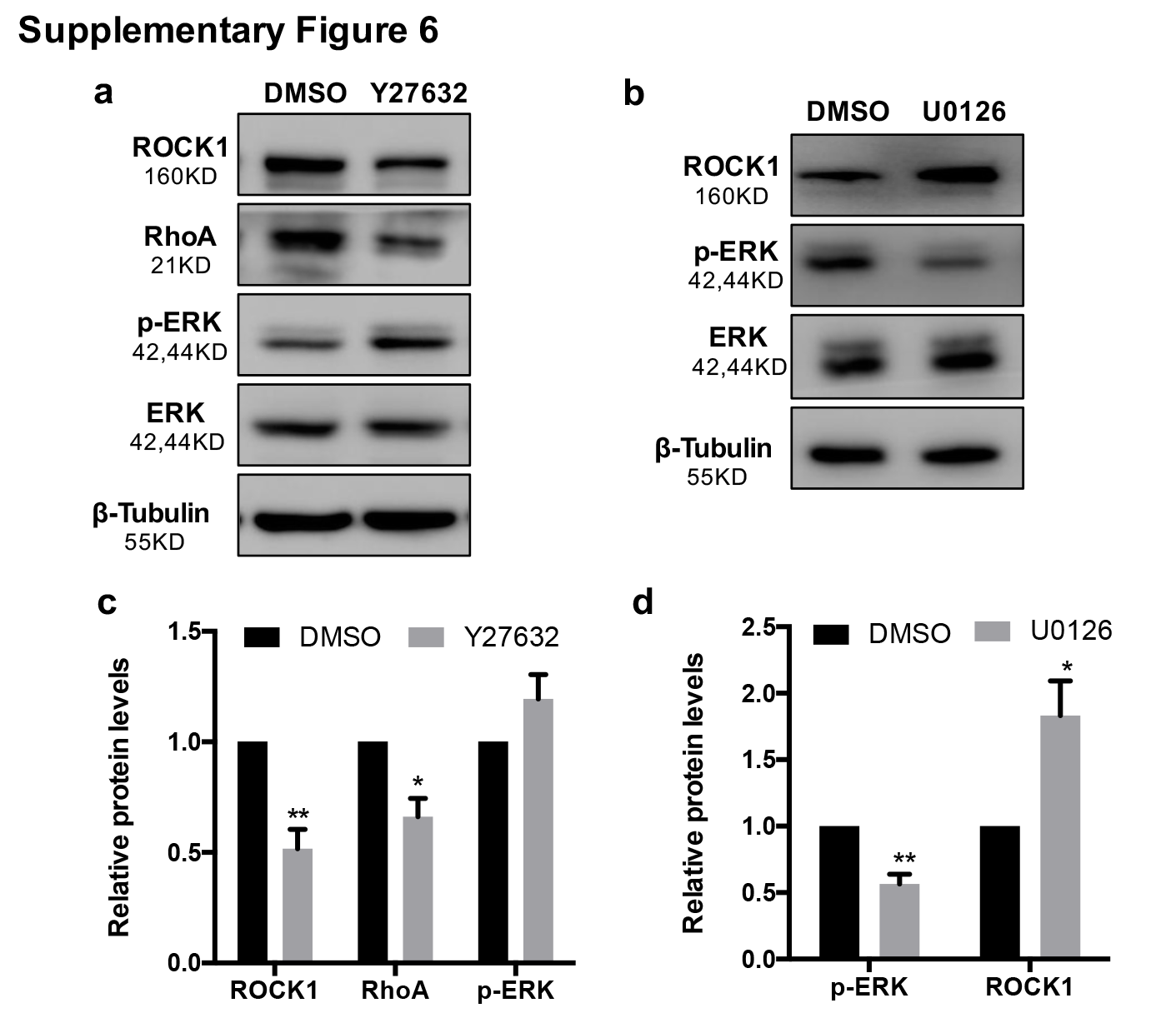

Supplement: Supplementary file 15 [file Image_6.tif]
